# Coupled on-line *in crystallo* UV–Vis absorption spectroscopy and X-ray crystallography to compare specific radiation damage in metal-containing proteins at room versus cryogenic temperature

**DOI:** 10.1107/S2059798326000690

**Published:** 2026-02-05

**Authors:** Nicolas Caramello, Samuel L. Rose, Eric Mathieu, Lucas Petit, Ivo Tews, Sylvain Engilberge, Antoine Royant

**Affiliations:** a Université Grenoble Alpes, CNRS, CEA, Institut de Biologie Structurale 38044Grenoble France; b Department of Chemistry National Taiwan University 1 Roosevelt Road Section 4 Taipei106 Taiwan; c European Synchrotron Radiation Facility 38043Grenoble France; d School of Biological Sciences University of Southampton Institute for Life Sciences (IfLS) B85 SouthamptonSO17 1BJ United Kingdom; University of Oxford, United Kingdom

**Keywords:** *in crystallo* UV–Vis absorption spectroscopy, room-temperature X-ray crystallography, specific radiation damage, metal-containing proteins

## Abstract

Specific radiation damage (SRD) to proteins is a pertinent issue discovered duringthe development of cryo-crystallography at synchrotrons, often affecting the macromolecular active site and thus complicating the understanding of mechanistic insights from structural analysis. For proteins with a spectroscopic signature in the visible light spectrum, *in crystallo* UV–Vis absorption spectroscopy has regularly been used to estimate the dose scale of specific damage build-up and to develop diffraction data-collection strategies to mitigate its effects. Using a coupled spectroscopic and crystallographic approach, here we show that for two metal-containing proteins the structural response to X-ray-induced reduction of metals in their active site is markedly different at room temperature than at cryogenic temperature. This suggests that the use of controlled specific radiation damage to mimic and study a physiological redox transition in a metal-containing protein by X-ray crystallography should preferably be performed at room temperature rather than at cryogenic temperature.

## Abstract 2

We used on-line *in crystallo* UV–Vis absorption spectroscopy in conjunction with X-ray crystallography on beamline BM07-FIP2 at the ESRF to compare the structural effects of specific radiation damage on two different metal-containing proteins at either room or cryogenic temperature.

## 1. Introduction

Radiation damage is an issue inherent to X-ray crystallo­graphy that affects the measured intensities of reflections, primarily resulting in their dose-dependent decay. In the early days of macromolecular X-ray crystallography (MX), this meant that diffraction data had to be collected from several crystals in order to obtain a complete dataset at room temperature (RT). The advent of cryo-crystallography (Hope *et al.*, 1989 ▸; Garman & Schneider, 1997 ▸) allowed the extension of the crystal lifetime in the X-ray beam by up to two orders of magnitude (Nave & Garman, 2005 ▸) and enabled a surge of structure determinations at synchrotrons. However, it became obvious that specific groups of the protein were affected by X-rays, such as disulfide bonds and carboxyl groups of acidic residues (Burmeister, 2000 ▸; Ravelli & McSweeney, 2000 ▸; Weik *et al.*, 2000 ▸). It was realized early on that X-rays could reduce the metal centres of metal-containing proteins at cryogenic temperatures (CTs), but it was suggested that the overall protein structure was likely to remain unchanged between different oxidation states (Lamb *et al.*, 1998 ▸). The subtle structural changes to chemical groups within the protein molecules were termed ‘specific’ radiation damage, while the decay in diffraction intensities, coupled with an increase in cell volume, Wilson *B* factor and often mosaicity, were named ‘global damage’ and largely affected the overall crystal lattice.

The difficulty in identifying and quantifying specific damage in electron-density maps called for the identification of orthogonal techniques that could serve to derive metrics for radiation damage. A seminal result was the identification of a peak at ∼400 nm in the UV–Vis absorption spectrum of an irradiated crystal of acetylcholinesterase ascribed to the formation of disulfide radicals, using an off-line microspectrophotometer (Weik *et al.*, 2002 ▸), prompting the development of on-line UV–Vis absorption microspectrophotometry at synchrotrons (McGeehan *et al.*, 2009 ▸). Other optical spectroscopy techniques were later implemented: Raman and fluorescence emission spectroscopy (Adam *et al.*, 2009 ▸). For metal-containing proteins, X-ray-based spectroscopies can also be applied as complementary techniques (Makita *et al.*, 2023 ▸): X-ray absorption near-edge spectroscopy (XANES) and extended X-ray absorption fine-structure (EXAFS) spectroscopy (Yano *et al.*, 2005 ▸), as well as X-ray emission spectroscopy (XES; Fransson *et al.*, 2018 ▸).

Here, we focus on single-crystal rotation X-ray crystallo­graphy experiments resulting in datasets during which specific damage progressively develops, and can also be termed multiple structures from one crystal (MSOX). This is in contrast to the classic serial crystallography (SX) approach, in which single still patterns obtained from several thousands of crystals form a dataset with homogenous specific damage. In a hybrid rotation/serial approach, complete datasets are constructed from several partial rotation datasets obtained from a number of different crystals (MSSX: multiple structures from several crystals). An MSOX study of comparative radiation damage at RT versus CT surprisingly showed that specific radiation damage was not detectable in electron-density maps, suggesting a coupling between global and specific damage at RT, while the two types are largely uncoupled at CT (Gotthard *et al.*, 2019 ▸). Raman and UV–Vis absorption spectroscopy techniques were used to show that disulfide-bond reduction, and decarboxylation of a critical acidic residue, did however occur within the crystal upon irradiation. The absence of visible specific damage can be explained by the fact that the build-up of the modified chemical group is concurrent with the loss of diffraction resolution on a rapid dose scale, progressively increasing the signal-to-noise ratio of the data required to observe the damage. This hypothesis was reinforced by an MSSX study showing the build-up of specific radiation damage on disulfide bonds at RT (de la Mora *et al.*, 2020 ▸). The use of several tens of crystals led to an increase in the signal to noise of the reconstructed datasets, likely explaining the difference in specific damage visibility. The logical follow-up to the MSOX study was to investigate specific damage at RT on metals, which are more electron-rich chemical groups and are usually more sensitive to X-rays than sulfur/oxygen/carbon-containing groups (Beitlich *et al.*, 2007 ▸).

MSOX radiation-damage studies performed at CT on metalloproteins have been numerous and essentially concluded on a subtle, yet significant alteration of the direct coordination sphere of the metal ion (Schlichting *et al.*, 2000 ▸; Berglund *et al.*, 2002 ▸; Adam *et al.*, 2004 ▸; Hersleth *et al.*, 2007 ▸; Gudmundsson *et al.*, 2014 ▸; Zárate-Romero *et al.*, 2019 ▸; Pfanzagl *et al.*, 2020 ▸), reorganization of the active site (Taberman *et al.*, 2019 ▸) or even catalytic turnover (Horrell *et al.*, 2016 ▸). We chose two metalloproteins for which specific radiation-damage effects have been well studied at CT, myoglobin (Hersleth *et al.*, 2007 ▸; Hersleth & Andersson, 2011 ▸; Pompidor *et al.*, 2013 ▸; Owen *et al.*, 2017 ▸; Pfanzagl *et al.*, 2020 ▸) and copper-containing nitrite reductase (Horrell *et al.*, 2016 ▸), first to see whether specific damage could be clearly visualized at RT in electron-density maps. Furthermore, we sought to compare the structural response of the protein at the two different temperatures, aided by UV–Vis absorption spectroscopy, allowing the unambiguous assessment of the redox states of the metal ions, which have distinctive spectroscopic signatures.

## 2. Methods

### 2.1. Protein preparation and crystallization

Horse-heart myoglobin (hhMb) was purchased from Sigma–Aldrich (product No. M1882). Copper-containing nitrite reductase from *Achromobacter cycloclastes* (*Ac*NiR) was produced and purified as described previously (Halsted *et al.*, 2019 ▸), with the protein buffer-exchanged into 10 m*M* MES pH 6.5 prior to crystallization. Both proteins were crystallized in-house under conditions optimized to yield single crystals suitable for X-ray diffraction. For myoglobin, the protein was resuspended at a concentration of 100 mg ml^−1^ in 100 m*M* sodium phosphate pH 7.8 buffer before being crystallized using the hanging-drop vapour-diffusion method at RT (20°C) with a precipitant solution consisting of 4 *M* sodium malonate pH 6, yielding rod-shaped crystals with a typical size of 170 × 40 × 30 µm (Supplementary Figs. S1*a* and S1*c*). The precipitant solution was also used as a cryoprotectant for data collection at 100 K. Bipyramid-shaped crystals of *Ac*NiR with an average size of 150 × 140 × 80 µm (Supplementary Figs. S1*b* and S1*d*) were obtained using the hanging-drop vapour-diffusion method at RT (20°C) with a precipitant solution consisting of 1.2 *M* ammonium sulfate, 50 m*M* citrate pH 4.75. Prior to data collection, crystals were soaked in a storage buffer consisting of 2.5 *M* ammonium sulfate, 50 m*M* citrate pH 4.8, with the addition of 17.5% sucrose as a cryoprotectant for data collection at 100 K.

### 2.2. Beamline setup and data collection

**Figure 1:**
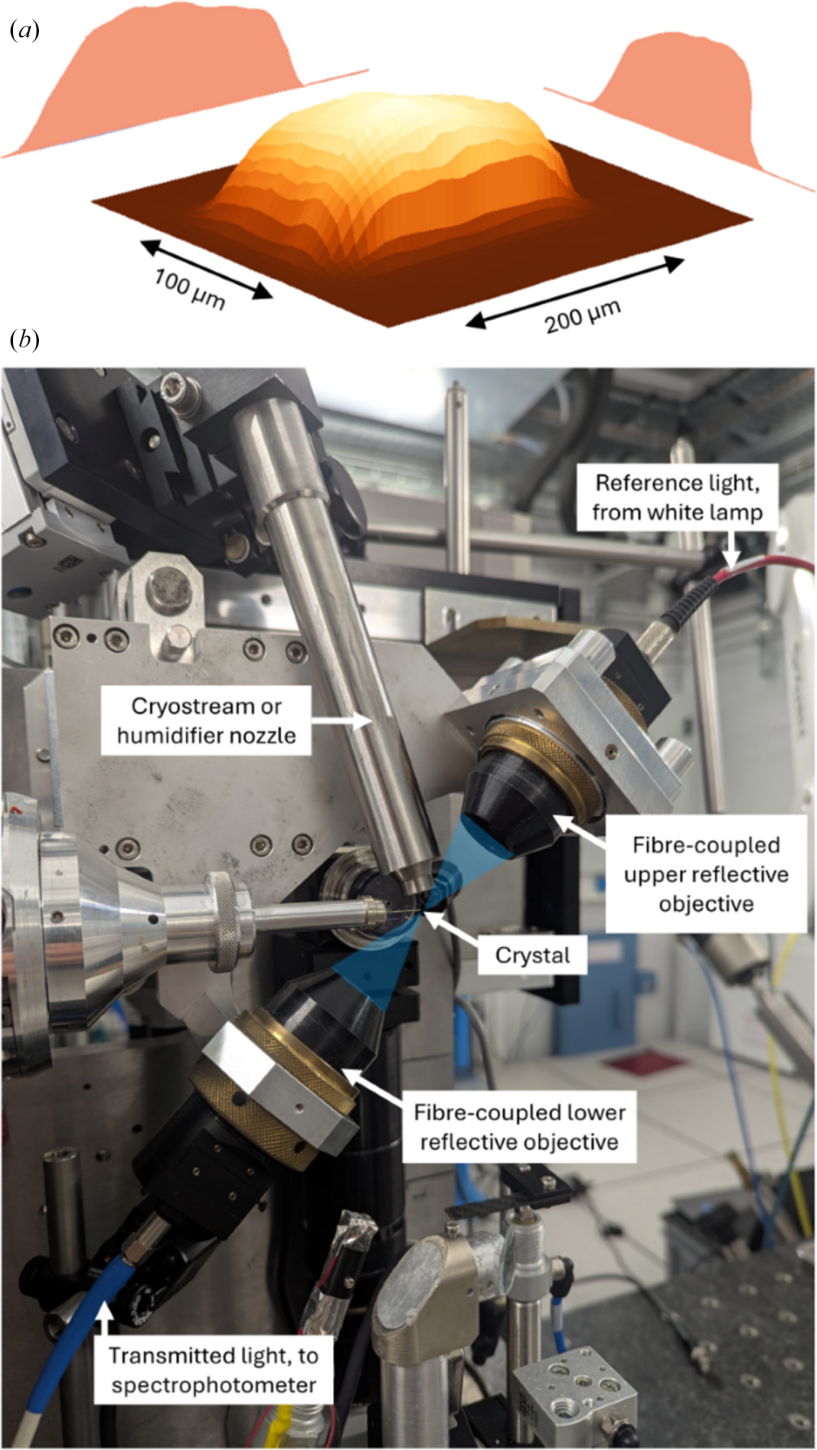
Experimental setup on beamline BM07 at the ESRF. (*a*) X-ray beam profile optimized for a 200 × 100 µm aperture at 12.658 keV. (*b*) On-line microspectrophotometer mounted on the beamline diffractometer. Key elements are labelled.

X-ray diffraction and spectroscopic measurements were performed on the BM07 beamline at the ESRF, Grenoble (McCarthy *et al.*, 2025 ▸). BM07 delivers a monochromatic X-ray beam with a top-hat profile (Fig. 1 ▸*a*). Data were collected at an energy of 12.658 keV using a PILATUS2 6M detector. Crystals were harvested and mounted directly on the minidiffractometer of BM07 either under cryogenic conditions (100 K) in the cold nitrogen stream of a 1000 series cryostream (Oxford Cryosystems) or at RT (294 K) using the HC-Lab humidity-control device (Arinax) to prevent dehydration during measurement.

For *Ac*NiR, 40 consecutive datasets were collected at 100 K with resolutions ranging from 1.58 to 1.97 Å. Each dataset consists of 200 images measured sequentially on the same wedge of the crystal, using 0.5° oscillation, 0.05 s exposure time and a flux of 1.125 × 10^11^ photons s^−1^. The average dose (exposed region) (ADER) for a single dataset was calculated with *RADDOSE*-3*D* (Bury *et al.*, 2018 ▸) to be 33 kGy. At RT, a single dataset of 4000 images was collected using 0.5° oscillation, 0.05 s exposure and a flux of 0.5 × 10^11^ photons s^−1^. This data-collection strategy was used to minimize crystal movement. This large dataset was subsequently subdivided into 19 sub-datasets of 200 images each (the final 200 images were unusable) with resolution ranging from 1.60 to 2.11 Å. The dose per 200-image dataset was calculated to be 15 kGy.

For myoglobin, 47 datasets were collected at 100 K, with a constant resolution of 1.07 Å. Each dataset consisted of 360 images measured using 0.5° oscillation, 0.05 s exposure and a flux of 4.95 × 10^10^ photons s^−1^. The dose for a single dataset was calculated with *RADDOSE*-3*D* to be 14.4 kGy. At RT, 18 datasets of 720 images each were measured successively on the same crystal using 0.5° oscillation, 0.1 s exposure and a flux of 2.11 × 10^10^ photons s^−1^. The dose for a single dataset was calculated to be 32.5 kGy, with resolutions ranging from 1.24 to 2.82 Å.

### 2.3. Beam characterization and flux measurement

The BM07 X-ray beam has a top-hat intensity distribution with a variable size between 200 × 100 µm (Fig. 1 ▸*a*) and 250 × 250 µm. The beam profile at the sample position was mapped at 12.658 keV using a 5 µm tungsten pinhole. The pinhole was translated along a surface of 300 µm in width and 200 µm in height with 50 points of measurement in each direction. A photodiode placed behind the pinhole recorded the transmitted intensity at each point. The integrated intensities were plotted in two dimensions using an in-house Python 3 script employing standard scientific plotting libraries (Matplotlib, SciPy, NumPy). The resulting 3D beam-intensity map (Fig. 1 ▸*a*) confirmed the top-hat distribution. The flux at the sample position was determined with a calibrated Canberra photodiode (model PD300-500CB) and estimated to be maximally 2.25 × 10^11^ photons s^−1^ for a beam size of 100 × 200 µm and 100% transmission.

### 2.4. *In crystallo* UV–Vis absorption spectroscopy

*In crystallo* UV–Vis absorption spectra were recorded using a microspectrophotometer installed in the sample environment of BM07 (McGeehan *et al.*, 2009 ▸; von Stetten *et al.*, 2015 ▸; Fig. 1 ▸*b*). A 400 µm optical fibre connected a white-light source (DH2000BAL, Ocean Optics) to the upper 4× reflective objective of the microspectrophotometer, while the lower 4× reflective objective was coupled to a QE-PRO spectrophoto­meter (Ocean Optics) via a 600 µm fibre. The same experimental setup was used to probe the effects of X-rays on various typical crystal-buffer and cryoprotecting components at CT (Stubbs *et al.*, 2026 ▸). Measurements were performed on crystals mounted on the diffractometer under the same conditions as for X-ray data collection. For each sample, the crystal position was optimized to improve the spectroscopic signal in the UV–Vis region and to minimize the baseline. Spectra were recorded continuously on a still crystal. After the collection of a number of spectra in the absence of X-rays, the X-ray shutter was opened to allow the evolution of the UV–Vis spectroscopic features of the metal centres to be monitoried as a function of accumulated dose. The characteristic absorption bands of the haem cofactor in myoglobin and of the T1Cu copper centre in its oxidized (Cu^2+^) state in green *Ac*NiR were used as initial markers to follow radiation-induced redox changes. Series of spectra were collected under both CT and RT conditions. Spectroscopic data were analysed and represented with the *ic*OS toolbox (Caramello *et al.*, 2025 ▸).

### 2.5. Dose calculations

Absorbed doses were calculated using *RADDOSE*-3*D* v.4.0.1020. The beam size was chosen so that every crystal was fully bathed in the X-ray beam. The program was used to calculate the average dose over the exposed region (ADER) for each individual dataset and to estimate the cumulative dose for the entire dataset series. All parameters, including beam size, photon flux, exposure time and rotation range, were defined according to the experimental settings. Dose values are expressed in kGy. For a given dataset, we use the term ‘effective absorbed dose’ to represent the sum of the dose absorbed during all previous data collections and half of the dose absorbed during this data collection, to take into account the fact that the latter dose is progressively accumulated during the course of the dataset.

### 2.6. X-ray data processing and refinement

Diffraction data were processed using the *autoPROC* pipeline (Vonrhein *et al.*, 2018 ▸), which integrates data with *XDS* and scales with *AIMLESS* and *TRUNCATE* from the *CCP*4 suite (Agirre *et al.*, 2023 ▸). Structures were refined using *REFMAC*5 (Yamashita *et al.*, 2023 ▸). Model inspection and manual building were performed in *Coot* (Emsley *et al.*, 2010 ▸). Difference electron-density maps (*F*_obs(*n*)_ − *F*_obs(1)_) were generated to visualize radiation-induced changes using the *phenix.fobs_minus_fobs* tool of the *Phenix* suite (Liebschner *et al.*, 2019 ▸). Final refined coordinates and structure factors have been deposited in the Protein Data Bank (Supplementary Tables S1, S2, S3 and S4).

In order to assess the iron-to-proximal histidine distance and the out-of-plane displacement of the haem iron in hhMb crystals (Supplementary Figs. S2*c* and S2*d*), hhMb structures were refined using an altered version of the library file describing the geometries of the haem and of its coordination bond to the proximal histidine. In this file, the parameters regulating the haem–His93 bond (the length of the Fe—N bond and the angles between the four pyrrole N atoms, Fe and the histidine N atom, as well as the corresponding dihedral angles) had their standard deviations increased significantly (up to 15% of the bond length/angle/dihedral values) to specifically relax the corresponding chemical restraints. The restraints on angles and dihedral angles regulating the planarity of the haem were similarly relaxed.

### 2.7. Difference density integration

To establish the kinetics of difference electron-density signals with dose (Fig. 3*b*), specific Fourier difference map features were integrated using in-house Python scripts, utilizing the *GEMMI* Python package (Yamashita *et al.*, 2023 ▸; available at https://github.com/ncara/Density-integration). The standard deviation σ of each map was extracted using the *MAPDUMP* tool from the *CCP*4 package (Agirre *et al.*, 2023 ▸). Difference structure factors were then used to re-calculate all maps with an identical sampling grid via the *GEMMI* package, and all positive or negative electron-density values above 3σ from voxels within a 1.0 Å radius of the atom of interest were integrated.

## 3. Results

### 3.1. Effects of X-ray-induced reduction on horse-heart myoglobin

Myoglobin is an oxygen-storage haem protein. In an oxygen-containing atmosphere, the protein is found in a hexacoordinated high-spin ferric (Fe^3+^) state with a water molecule bound to the haem iron (Lamb *et al.*, 1998 ▸), termed metmyoglobin (metMb). Here, we assessed the effect of X-rays on the electronic environment of the haem iron of horse-heart myoglobin (hhMb) at CT and RT using on-line UV–Vis absorption spectroscopy. We then compared the structural response of the protein to X-ray-induced reduction of the haem iron in a dose-resolved manner at CT, and at RT, via sequentially collected MSOX datasets.

#### 3.1.1. Tracking the electronic state of haem iron with dose-resolved on-line *in crystallo* UV–Vis absorption spectroscopy

The UV–Vis absorption spectrum of hhMb crystals at CT features a highly absorbing Soret band at 410 nm (peak in the blue spectrum highlighted by a dashed line; Supplementary Fig. S2*a*), a strong Q_v_ band at 500 nm and hints of weaker Q_v_ bands at 525 and 570 nm (plain arrows; Supplementary Fig. S2*a*), and finally a large Q_0_ band centred at 630 nm, indicating that the protein is in the metMb state.

**Figure 2:**
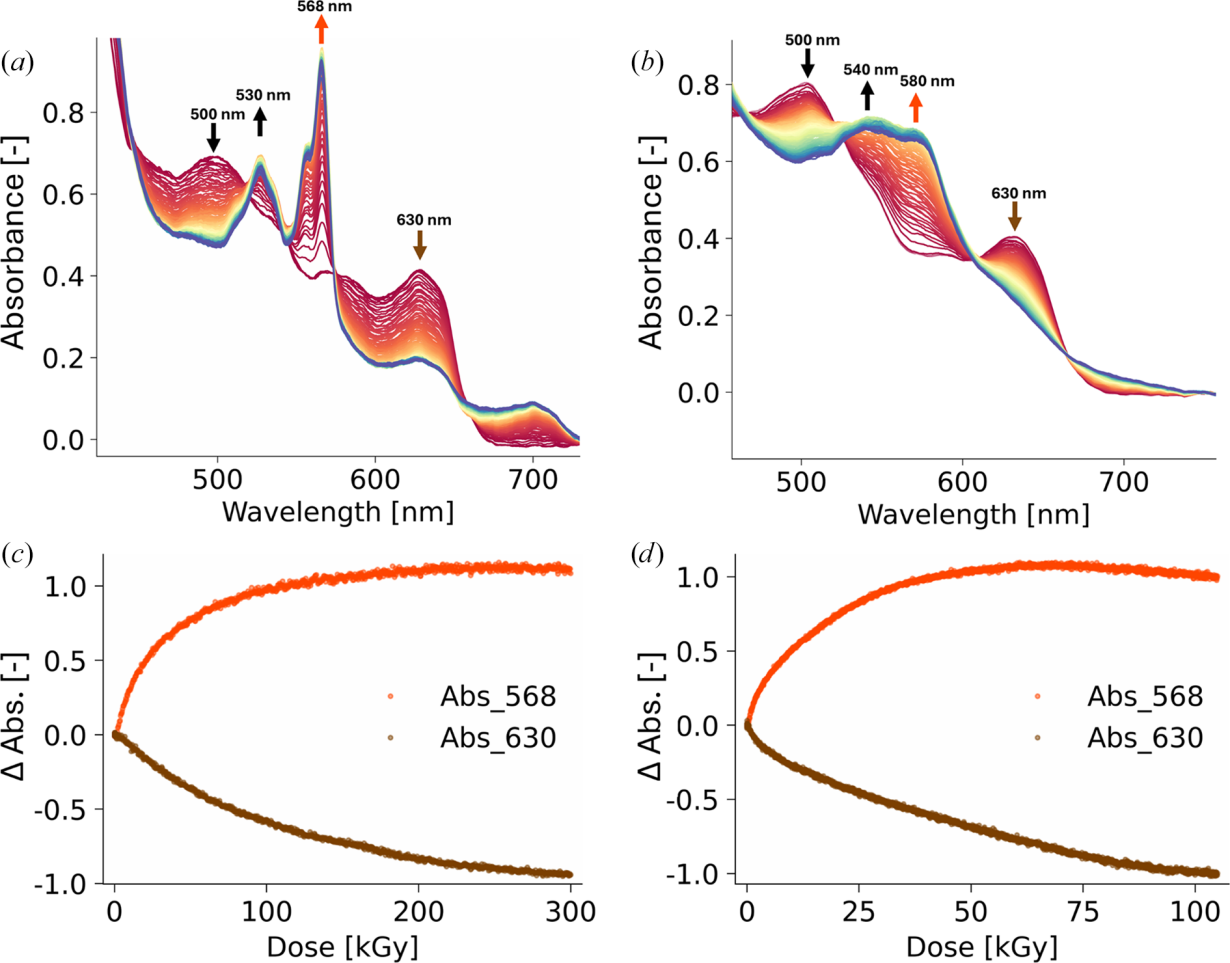
X-ray-induced reduction of hhMb followed by on-line *in crystallo* UV–Vis absorption spectroscopy. (*a*) Dose-resolved series of CT spectra, from red (0 kGy) to blue (2 MGy). (*b*) Equivalent dose-resolved series at RT over a shorter dose range for readability, from red (0 kGy) to blue (115 kGy). (*c*) Evolution of absorbance at 568 nm (red) and 630 nm (brown) as a function of dose at CT. The dose constants were calculated to be 30 and 80 kGy, respectively. (*d*) Evolution of absorbance at 568 nm (red) and 630 nm (brown) as a function of dose at RT. The reduction dose constants were calculated to be 19 and 48 kGy, respectively.

Upon progressive X-ray-induced reduction, the wide Q_v_ band splits, forming sharp peaks at 515, 535, 545 and 568 nm, while the Q_0_ band decays (Fig. 2 ▸*a*), in good agreement with previous observations (Beitlich *et al.*, 2007 ▸). The 568 nm Q_v_ band is associated with a reduced, low-spin ferrous (Fe^2+^) haem iron that is still hexacoordinated (Hersleth *et al.*, 2007 ▸), while the Q_0_ band is specific to the Fe^3+^ metMb state (Hersleth *et al.*, 2008 ▸). As was also previously noted (Hersleth & Andersson, 2011 ▸), the 568 nm Q_v_ band rises faster [dose constant (here, rise to 1/*e* of the asymptotic value) of 30 kGy, red trace in Fig. 2 ▸*c*] than the 630 nm Q_0_ band decays [dose constant (here, decay to 1/*e* of the initial value) of 80 kGy, brown trace in Fig. 2 ▸*c*]. The Soret band also red-shifts to 425 nm upon reduction (Supplementary Fig. S2*b*).

The observed decoupling of band-evolution kinetics suggests that the haem iron is reduced first from Fe^3+^ to Fe^2+^, faster than the haem electronic structure and coordination environment can relax. The decay of the Q_0_ band then leads to the spectroscopic signature of a water-coordinated low-spin Fe^2+^ state, as previously observed after X-ray-induced reduction at CT (Beitlich *et al.*, 2007 ▸; Hersleth *et al.*, 2007 ▸).

The UV–Vis absorption spectrum of metMb crystals at RT closely resembles that recorded at CT, with all band maxima positioned at almost the same wavelengths (orange spectrum; Supplementary Fig. S2*a*). Upon progressive X-ray-induced reduction, in contrast to the cryogenic condition, two wide, strong Q_v_ bands rise maximally at 525 and 580 nm (Fig. 2 ▸*b*) with a dose constant of 19 kGy (Fig. 2 ▸*d*), while the Q_0_ band decays with a dose constant of 48 kGy, reminiscent of the rate decoupling observed in the CT series. Above 110 kGy, only the 540 and 580 nm Q_v_ bands remain (Supplementary Fig. S2*b*), showing that the crystals have been fully converted to the pentacoordinated high-spin ferrous (Fe^2+^) Mb state (or deoxyMb) state, as previously characterized (Lamb *et al.*, 1998 ▸). Altogether, this strongly suggests that upon X-ray-induced reduction the haem iron immediately loses its coordinating water molecule (rise of the 540 and 580 nm Q_v_ bands) before the haem can adapt to this new state by moving from a metMb to a deoxyMb geometry (decay of the 630 nm Q_0_ band). The Soret band also red-shifts to 425 nm upon reduction (Supplementary Fig. S2*b*).

#### 3.1.2. Differential structural response of hhMb to X-ray-induced reduction of the haem iron between CT and RT

**Figure 3:**
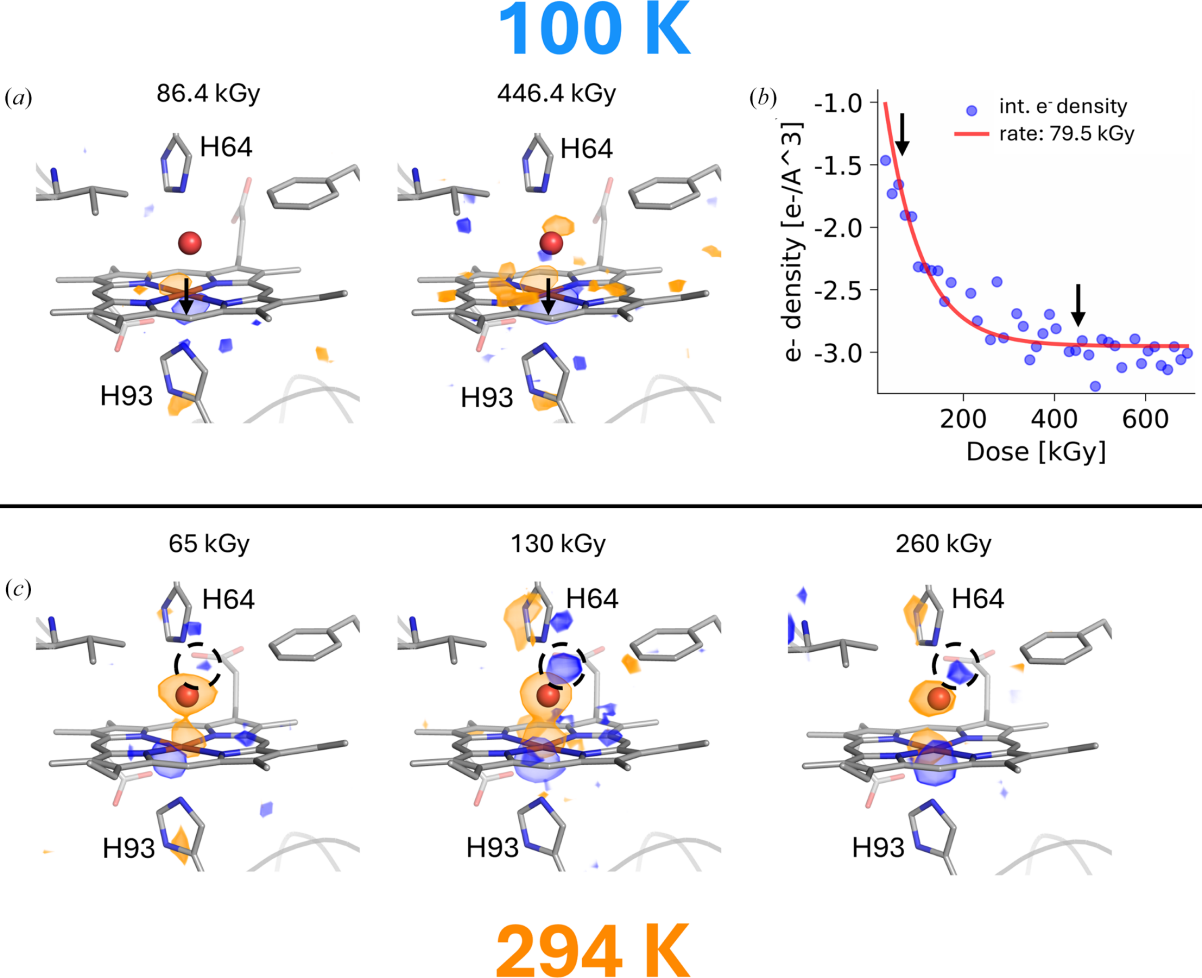
Structural response of hhMb to X-ray-induced reduction. (*a*) *F*_obs(*n*)_ − *F*_obs(1)_ Fourier difference maps corresponding to dose points 86.4 and 446.4 kGy at CT contoured at a ±3.5σ level (orange, negative; blue, positive) and superimposed on the refined 14.4 kGy model (PDB entry 9t6y). (*b*) Integrated negative difference electron density at the location of the haem iron as a function of dose, building up with a dose constant of 79.5 kGy. (*c*) *F*_obs(*n*)_ − *F*_obs(1)_ Fourier difference maps corresponding to dose points 65.0, 130.0 and 260.0 kGy at RT contoured at a ±3.5σ level (orange, negative; blue, positive) and superimposed on the refined 32.5 kGy model (PDB entry 9t6v).

The structure of metMb was determined at CT with an absorbed dose of 14.4 kGy (PDB entry 9t6y), equivalent to an effective dose of 7.2 kGy, corresponding to 15% of the fastest reduction dose constant determined by UV–Vis absorption spectroscopy, thus demonstrating an oxidized state of the iron. The Fe atom is hexacoordinated, with a water molecule as its sixth coordination point (grey structure in Fig. 3 ▸*a*, beige structure in Supplementary Fig. S3*a*). 47 datasets were sequentially collected from the same crystal, producing dose snapshots of increasingly reduced Mb, with a dose resolution of 14.4 kGy. The isomorphous difference electron-density map calculated between the sixth and the first dataset [*F*_obs_(86.4 kGy) − *F*_obs_(14.4 kGy)] already features a strong negative peak above the iron in Fig. 3 ▸(*a*) (−4.4σ or 0.129 e^−^ Å^−3^), with a corresponding positive peak of comparable strength (+4.8σ or 0.137 e^−^ Å^−3^) below the iron (Fig. 3 ▸*a*). The magnitudes of the two peaks only increase with dose (see Fig. 3 ▸*b* for the evolution of the negative peak), with very similar dose constants of ∼80 kGy, close to the dose constant of the Q_0_ band decay. At higher doses (above 400 kGy), a similar pair of peaks appear on either side of the coordinated water molecule, as well as negative peaks above the four N atoms of the haem (Fig. 3 ▸*a*). Structural refinement of a single-conformation model to successive datasets with relaxed restraints on the haem geometry show a progressive decrease in the distance between the iron and the coordinating N atom of the proximal histidine His93 (Supplementary Fig. S2*d*). In the final, reduced state (676.8 kGy; PDB entry 9t6x; red structure in Supplementary Fig. S3*a*), as shown by the UV–Vis absorption spectra, both the haem and the coordinated water molecule have shifted in a concerted way, maintaining the hexacoordinated geometry of the iron.

The equivalent experiment was performed at RT. 18 datasets were sequentially collected from the same crystal, producing dose snapshots of increasingly reduced Mb, with a dose resolution of 32.5 kGy. The effective absorbed dose of the first structure (PDB entry 9t5v) is 32.5/2 = 16.3 kGy, which corresponds to 86% of the fastest reduction dose constant measured by UV–Vis absorption spectroscopy (Fig. 2 ▸*d*), indicating that the haem iron has been significantly reduced, in contrast to the first structure of the CT series. According to the red curve in Fig. 2 ▸(*d*), the 16.3 kGy dose point corresponds to an average structure with approximately 50% ferrous and 50% ferric iron. When compared with the lower dose CT structure (Supplementary Fig. S2*e*), the lower dose RT structure has its iron significantly displaced out of the haem plane (Supplementary Figs. S2*c* and S2*e*), increasing its distance to the coordinating water molecule (2.12 versus 2.06 Å, respectively; Supplementary Figs. S2*d* and S2*e*). Residues in the haem-binding pocket exhibit small outward displacements, which are likely not to have occurred upon iron reduction only, but to have arisen from protein contraction upon flashcooling, as previously observed for myoglobin (Frauenfelder *et al.*, 1991 ▸) and other proteins (Fraser *et al.*, 2011 ▸).

The structural response of hhMb to X-ray-induced iron reduction at RT is illustrated by the strong features present in the [*F*_obs_(65.0 kGy) − *F*_obs_(32.5 kGy)] difference map (Fig. 3 ▸*c*), suggesting displacement of the coordinating water molecule. As dose increases, the iron is further displaced out of the haem plane (Supplementary Figs. S2*c* and S3*b*) with a rate matching that of the Q_0_ band decay. A positive peak becomes dominant in the [*F*_obs_(130.0 kGy) − *F*_obs_(32.5 kGy)] difference map (dotted circle in Fig. 3 ▸*c*) at 1.85 Å from the position occupied by the water in the first structure. This peak arises from the relocation of the coordinating water molecule, as validated by the last usable structure of our series (absorbed dose of 260 kGy; PDB entry 9t6w). Accordingly, the side chain of His64 reorientates to maintain hydrogen bonding to the water molecule (Supplementary Fig. S3*b*). Structural refinement of a single-conformation model to successive datasets with relaxed restraints on the haem geometry reveals that the Fe atom progressively moves out of the porphyrin ring plane, while the plane itself deforms (‘doming-out’) as a structural response to the new coordination of the iron (Supplementary Figs. S2*c* and S3*b*). The new position of the water molecule as well as the dome-shaped geometry of the haem are characteristic of the deoxyMb state, as seminally obtained via cryotrapping crystallography (Lamb *et al.*, 1998 ▸).

### 3.2. Effects of X-ray-induced reduction on a copper-containing nitrite reductase (CuNiR)

The copper-containing nitrite reductase from *A. cycloclastes* (*Ac*NiR) in its substrate-free state was used as the second target to assess differences in X-ray-induced reduction at CT and RT. *Ac*NiR is a homotrimeric metalloenzyme which catalyses the reduction of its substrate nitrite (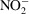) to nitric oxide (NO) (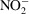 + 2H^+^ + e^−^ → NO + H_2_O). It contains two types of copper metal centres per monomer: an electron-accepting/donating type 1 copper (T1Cu) centre and a catalytic type 2 copper (T2Cu) centre. The binding of the chemical substrate occurs via displacement of a solvent ligand(s) [water (H_2_O) or hydroxide (OH^−^)] found in its resting state, and its reduction takes place following inter-copper electron transfer (ET) from T1Cu to T2Cu (∼12.5 Å away) through a conserved Cys–His bridge. It is already known that X-rays can rapidly reduce the primary T1Cu redox centre in CuNiRs from T1Cu^2+^ to T1Cu^+^, with this allowing redox-induced changes to subsequently occur at the T2Cu active site (Hough *et al.*, 2008 ▸; Horrell *et al.*, 2016 ▸). Several studies have used this phenomenon to initiate enzyme turnover in substrate-soaked crystals using the MSOX approach in *Ac*NiR and CuNiRs from *Bradyrhozobium* species (Horrell *et al.*, 2016 ▸, 2018 ▸; Hough *et al.*, 2020 ▸; Rose *et al.*, 2022 ▸, 2024 ▸). The valence state of T1Cu can also be monitored by UV–Vis absorption spectroscopy due to the strong absorbance bands seen in the T1Cu^2+^ oxidized state that are lost upon reduction to T1Cu^+^.

#### 3.2.1. X-ray-induced reduction of the T1Cu redox site at CT and RT tracked by dose-resolved on-line *in crystallo* UV–Vis absorption spectroscopy

The T2Cu site in CuNiRs is optically silent, so only the T1Cu site of *Ac*NiR could be monitored spectroscopically by on-line UV–Vis absorption spectroscopy. The CT (100 K) absorption spectrum of oxidized *Ac*NiR has signature bands at ∼454, ∼566 and ∼385 nm for the T1Cu^2+^ site, respectively, which can be assigned as S(Cys) → Cu^2+^**σ**, S(Cys) → Cu^2+^**π** and S(Met) → Cu^2+^ ligand-to-metal charge-transfer (LMCT) transitions, and are characteristic of the green subtype of CuNiRs. A broad band at ∼620–800 nm is also present and can be assigned as *d*–*d* transitions (Supplementary Fig. S4*a*). On the other hand, the RT (294 K) absorption spectrum of *Ac*NiR is vastly different, with the higher energy 454 and 385 nm bands red-shifting slightly to 457 and 390 nm, respectively, with a clear reduction in intensity, and the lower energy band at ∼568 nm red-shifting further to ∼585 nm. The ratios between the two peak maxima for S(Cys) → Cu^2+^**π**, S(Cys) → Cu^2+^**σ** and S(Met) → Cu^2+^ LMCT transitions shift from 1.28 to 1.05, respectively. As a result, the redistribution of bands brings it closer to the greenish-blue subtype of CuNiRs, with a similar thermodynamic equilibrium between green and blue copper sites being observed previously (Ghosh *et al.*, 2009 ▸). At this temperature the broader band assigned to *d*–*d* transitions is also less noticeable.

**Figure 4:**
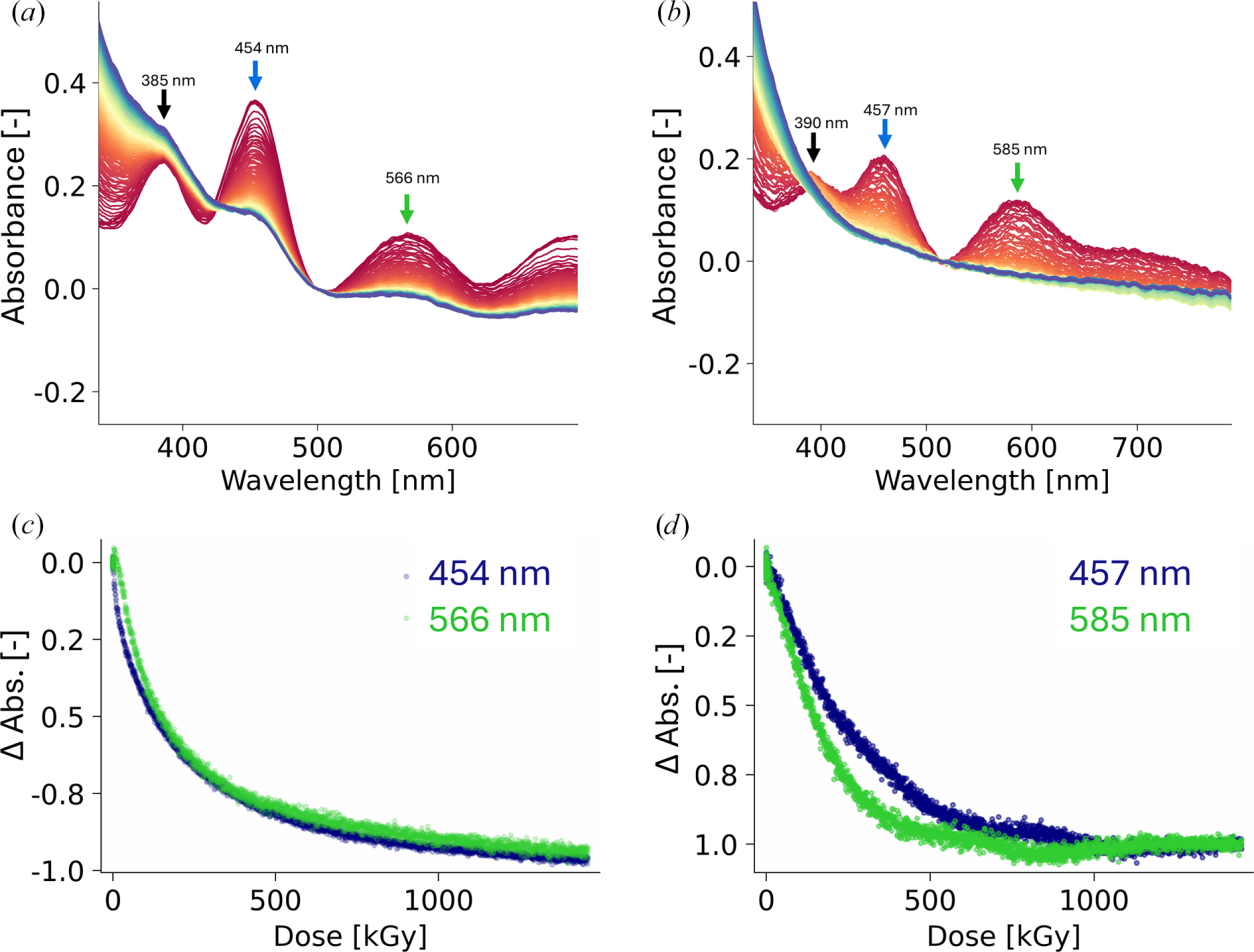
Comparison of the X-ray-induced reduction of T1Cu in *Ac*NIR crystals at CT and RT, monitored by on-line UV–Vis absorption spectroscopy. Series of UV–Vis absorbance spectra recorded during X-ray exposure from 0 (red) to 1.460 MGy (blue) at (*a*) CT and (*b*) RT. (*c*) Decay of absorbance at 454 nm (blue) and 566 nm (green) at CT as a function of dose; the decay dose constants were calculated to be 279 and 271 kGy, respectively. (*d*) Decay of absorbance at 457 nm (blue) and 585 nm (green) at RT as a function of dose; the decay dose constants were calculated to be 283 and 177 kGy, respectively.

Following X-ray-induced reduction of the T1Cu site, bleaching of all three bands is observed at both temperatures, which is an indication of a change in oxidation state to T1Cu^+^ (Fig. 4 ▸). The reduction of the ∼390 nm band is masked by a general baseline drift towards the near-UV region, but monitoring of the bands at ∼454/457 and ∼566/585 nm over the dose series at the two temperatures gives a variation in decay rates. At CT, the decay dose constants of the two bands (454 and 566 nm) are roughly the same (279 and 271 kGy, respectively), with similar decay dose constants seen for a substrate-free bluish-green *Bradyrhizobium* CuNiR at CT (∼585 nm decay; 330 kGy; Rose *et al.*, 2024 ▸). At RT, the two bands decay at different rates (Figs. 4 ▸*c* and 4 ▸*d*), with the 457 nm band decaying at a similar rate to the CT series (blue; Fig. 4 ▸*d*; 283 kGy) and the 585 nm band (green; Fig. 4 ▸*d*) decaying faster with a dose constant of 177 kGy. The faster decay rate of the 585 nm band in this series, assigned to S(Cys) → Cu^2+^**π** LMCT, shows a temperature-dependent electronic route of ET to T2Cu favouring blue T1Cu organization, which differs from what was previously proposed (Farver *et al.*, 2004 ▸; Rose *et al.*, 2024 ▸).

#### 3.2.2. Structural comparison of the T1Cu and T2Cu sites at CT and RT

Similar dose series of diffraction data were collected to provide a structural comparison at the different temperatures for substrate-free (*i.e.* as-isolated) *Ac*NiR. At the T1Cu site, which correlates with the spectroscopic signatures observed above, a slight difference can be observed between the structures derived from both starting datasets (CT, 33.3 kGy; PDB entry 9t6q; RT, 14.9 kGy, PDB entry 9t6o). The T1Cu coordination distances for the four coordinating ligands show a slight deviation between the two temperatures, agreeing with the temperature-dependent changes observed in the absorption spectra (Supplementary Fig. S4*a*). A slight elongation of the Cu—S(Met150) bond (2.45 to 2.53 Å) can be observed, together with a slight shortening of the Cu—S(Cys136) bond (2.21 to 2.18 Å), when the temperature is increased from CT to RT, with this consistent with a likely thermodynamic transition from green towards a bluish-green or blue subtype of CuNiR. Small variations in coordination distances are also observed for the other two coordinating ligands at CT and RT, respectively: Cu—N(His140), 1.95 Å versus 1.90 Å; Cu—N(His95), 2.02 versus 2.05 Å (Supplementary Table S5).

**Figure 5:**
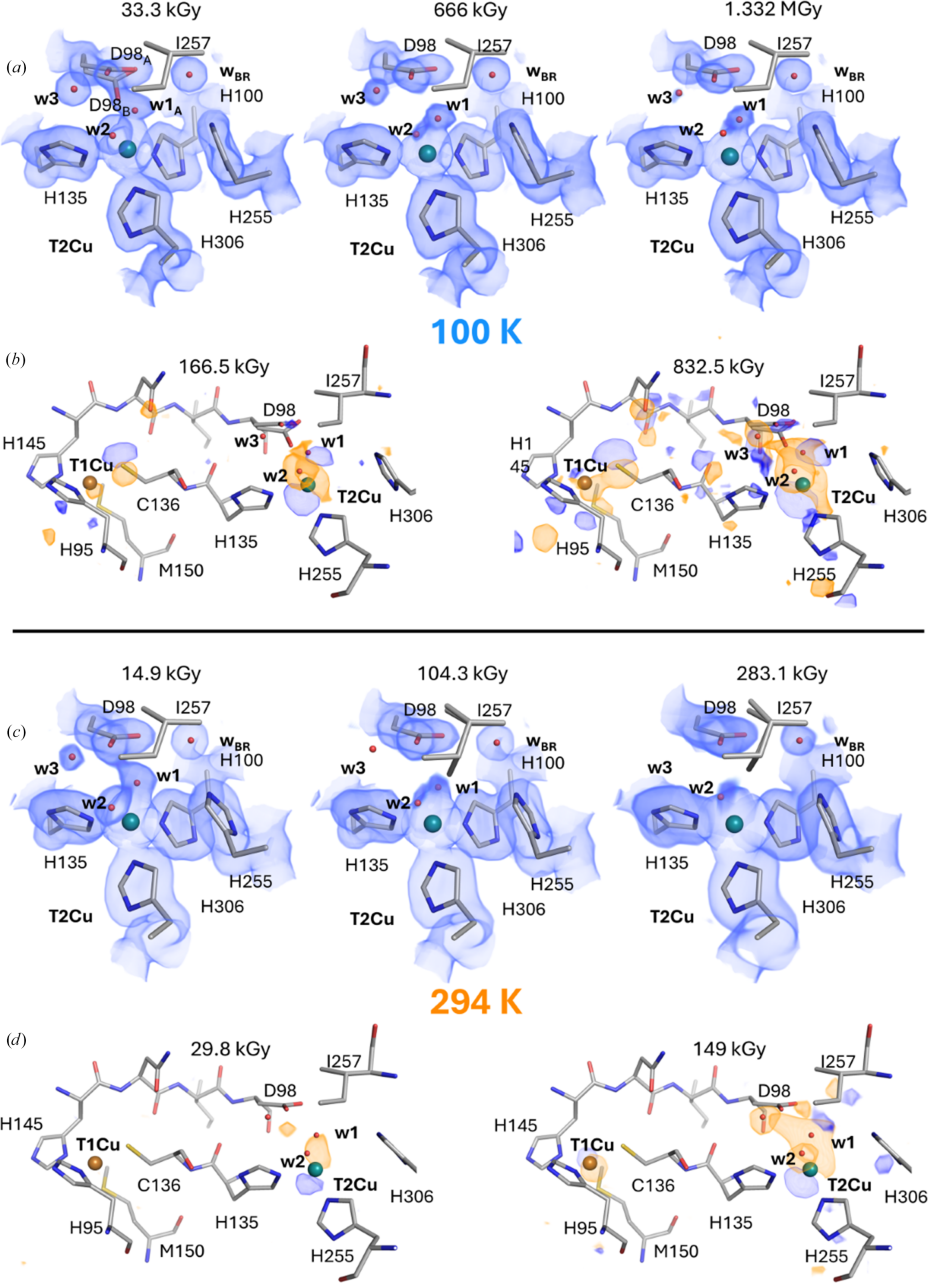
Structural response of *Ac*NIR to X-ray-induced reduction. (*a*) 2*F*_obs_ − *F*_calc_ electron-density map (blue) contoured at a 1.0σ level and superimposed on the corresponding refined structure (grey) at increasing absorbed doses [minimum (PDB entry 9t6q), medium and maximum (PDB entry 9t6u)] at CT. The bridging water molecule is labelled w_BR_. (*b*) *F*_obs(*n*)_ − *F*_obs(1)_ Fourier difference maps corresponding to dose points 166.5 and 832.5 kGy at CT contoured at a ±3.5σ level (orange, negative; blue, positive) and superimposed on the refined 33.3 kGy structure (PDB entry 9t6q). (*c*) 2*F*_obs_ − *F*_calc_ electron-density map (blue) contoured at a 1.0σ level and superimposed on the refined model (grey) at increasing absorbed doses [minimum (PDB entry 9t6o), medium and maximum (PDB entry 9t6p)] at RT. (*d*) *F*_obs(*n*)_ − *F*_obs(1)_ Fourier difference maps corresponding to dose points 29.8 and 149.0 kGy at RT contoured at a ±3.5σ level (orange, negative; blue, positive) and superimposed on the refined 14.9 kGy structure (PDB entry 9t6o).

At the T2Cu active site, marked differences can be observed between the two temperatures for the starting dataset. At CT a dual active-site conformation is present (Fig. 5 ▸*a*, Supplementary Figs. S5*a* and S5*b*), with one active-site conformation that is almost the same as a previous XFEL structure of substrate-free oxidized *Ac*NiR [also obtained at CT (100 K) using the serial femtosecond rotational crystallo­graphy (SF-ROX) approach at SACLA; Halsted *et al.* (2019 ▸)]. In the cryogenic XFEL structure at 1.60 Å resolution, a single T2Cu-coordinating water ligand could be modelled in a distorted tetrahedral geometry relative to the histidine plane. In addition, a catalytic aspartic acid residue (Asp98_CAT_), which sits within the active-site pocket and is usually orientated towards the T2Cu in a proximal position, was also seen to be partially rotated around the O^δ1^ atom in a new distorted conformation bringing it closer to the ligating water. The same features are also observed in our synchrotron-radiation (SR) cryogenic structure (PDB entry 9t6q), with the distorted position of Asp98_CAT_ similarly rotated 34° around the O^δ1^ atom (40% occupancy) with an ordered water ligand (w2; 100% occupancy) in a distorted tetrahedral geometry. However, a secondary active-site conformation is also present with the addition of a partial T2Cu-coordinating solvent ligand (w1), which can also be modelled (60% occupancy) in an active-site conformation with both the w2 ligand and the proximal position of Asp98_CAT_ (60% occupancy). In the RT structure (PDB entry 9t6o; Fig. 5 ▸*c* and Supplementary Fig. S5*c*), the dual active-site conformation is not observed, but instead a single active-site pocket configuration is seen with two full occupancy solvent ligands (w1 and w2) coordinated to T2Cu, together with Asp98_CAT_ in its proximal position only. This is the first time that a five-coordinated T2Cu site has been captured for any CuNiR at ambient temperatures and provides some evidence that a pentacoordinated T2Cu site could be the common coordination geometry for all CuNiRs in the T2Cu^2+^ valence state, with this also being observed in the major active conformation in the cryogenic structure and in other catalytically inefficient CuNiRs from *Bradyrhizobium* species (Rose *et al.*, 2021 ▸, 2022 ▸, 2024 ▸).

For the two dose series of substrate-free *Ac*NiR at the two temperatures, where X-ray-induced reduction of the primary T1Cu metal would be expected to initiate inter-Cu ET to T2Cu, several noticeable differences are also observed. 40 datasets (33.3 kGy per dataset) were sequentially collected from the same crystal for the CT series, while for RT 19 datasets were sequentially collected with a dose per dataset of 14.4 kGy. At the T2Cu active site at CT, the additional distorted conformation of the Asp98_CAT_ residue is slowly lost upon reduction, moving to a single proximal conformation (Figs. 5 ▸*a*, 5 ▸*b* and Supplementary Fig. S6*a*). By an absorbed dose of 660 kGy (Figs. 5 ▸*a* and 5 ▸*b*), this coincides with a partial loss of the ordered w2 solvent ligand from the active site (40% occupancy) and a geometrical shift of the partial w1 solvent ligand (60% occupancy) to a tetrahedral coordination with the T2Cu. Due to the contact distance between these two solvent ligands being too short (1.77 Å), it likely indicates two alternate conformations of a single T2Cu solvent ligand, with the tetrahedral position being the major one. This T2Cu active-site arrangement remains until the final dataset in the dose series (1332 kGy; PDB entry 9t6u). Throughout the dose series, the T2Cu atom also slowly drops into the histidine plane, moving 0.33 Å during the accumulated dose of 1332 kGy (Supplementary Fig. S4*c*), with this movement of T2Cu being a potential indication of T2Cu reduction from T2Cu^2+^ to T2Cu^+^, as observed in a chemically reduced structure of *Ac*NiR (Halsted *et al.*, 2019 ▸), where it dropped 0.5 Å. Around the active-site coordination sphere, the other catalytic residue, His255_CAT_, which has a proposed role in proton transfer together with Asp_CAT_ (Kataoka *et al.*, 2000 ▸), exhibits no signs of movement during the entire dose series, as with Ile257_CAT_, a residue shown to control ligand access to the T2Cu (Boulanger & Murphy, 2003 ▸; Fig. 5 ▸*b*).

At RT, similar movements of the T2Cu solvent ligands (w1 and w2) seen at CT can also be observed but at a much lower absorbed dose. By 104.3 kGy, a similar partial loss of the w2 solvent ligand (40% occupancy) coincides with a partial loss and shift of the w1 solvent ligand (50% occupancy) to a tetrahedral coordination with the T2Cu (Figs. 5 ▸*c*, 5 ▸*d* and Supplementary Fig. S6*b*). The half occupancies and short contact distance between these two solvent ligands (also 1.77 Å) confirms this as being two different active-site conformations with a single T2Cu solvent ligand in alternative positions. At this higher temperature, a minor active-site conformation (10% occupancy) also begins to appear with flipping of the C^δ1^ side chain of Ile257_CAT_ into the active-site pocket (Fig. 5 ▸*d*). This movement only occurs when the active-site pocket is empty and the T2Cu is tricoordinated and devoid of a ligand, representing a dead-end T2Cu^+^ state where no substrate can bind (Strange *et al.*, 1999 ▸). By the final dataset in the dose series with an absorbed dose of 283.1 kGy (PDB entry 9t6p), a dual active-site conformation exists with a single T2Cu solvent ligand (Wa; 55% occupancy) coordinated in a distorted tetrahedral geometry and an empty tricoordinated T2Cu site, with further flipping of the Ile257_CAT_ side chain into the pocket (40%; Figs. 5 ▸*c*, 5 ▸*d* and Supplementary Fig. S6*b*). His255_CAT_ also shows some signs of rotation of its imidazole ring during the dose series, effectively switching the hydrogen-bonding network from the carbonyl O atom of Glu279 (2.59 to 2.51 Å) to the hydroxyl O atom (O^1^) of Thr280 (3.03 to 2.67 Å) in a potential redox-coupled proton-transfer mechanism (Fukuda *et al.*, 2016 ▸).

At the T1Cu site, less obvious structural changes are observed at both temperatures, with variation in coordination distances and no interpretable trends (Supplementary Table S5). The Fourier difference maps reveal movement of the Cys136 S^γ^ atom at CT but not at RT and, similarly, movement of the T1Cu atom itself can also be observed in the RT series, with this not evident at CT. These suggest that there are some temperature-dependent dynamics taking place at the T1Cu site, but it is difficult to deduce true X-ray-induced changes from structure alone.

## 4. Discussion

We studied the comparative response of two paradigmatic metalloproteins of different types, one iron-containing and one copper-containing, to X-ray-induced metal reduction at CT and RT.

For hhMb, the structures obtained following X-ray-induced photoreduction of the haem iron differ in a few significant places at the two temperatures (Supplementary Fig. S3). Firstly, at CT the ferrous (Fe^2+^) iron is still hexacoordinated with a coordinating water molecule, while it is only pentacoordinated at RT, with the coordinating water molecule in the latter being able to relocate to a nearby position. Furthermore, at RT the haem deforms from its planar geometry into a dome-like structure, in which the Fe atom sits at the top. In contrast, at CT the whole haem remains planar but shifts as a whole within the haem-binding pocket towards the proximal histidine His93, suggesting that these structural rearrangements are hindered at CT.

For substrate-free *Ac*NIR, the structures obtained following X-ray-induced photoreduction of the T1Cu atom only show X-ray-induced changes at the T2Cu site, with the T1Cu site remaining largely structurally silent throughout the dose series (Supplementary Fig. S6). At the T2Cu site, structural rearrangement of the coordinating solvent ligands alters the overall coordination site, with an initial pentacoordination, suspected to be in a T2Cu^2+^ oxidized state at early dose values, evolving to a tetracoordinated site at CT, as a result of a coordinating solvent ligand vacating the active site. The results here suggest the vacating solvent ligand is w2, which is coordinated in a distorted tetrahedral geometry relative to the histidine plane. The remaining solvent ligand remains coordinated in a perfect tetrahedral geometry. Conversely, at RT the T2Cu site evolves from a pentacoordinated to a tetracoordinated site and eventually to a tricoordinated site, with the initial two solvent ligands both gradually vacating to leave the T2Cu devoid of any ligand and allowing the side chain of Ile257_CAT_ to flip into the vacant active site. The flipping of the Ile257_CAT_ side chain only at RT is also consistent with another similar study on *Ac*NiR (Sen *et al.*, 2017 ▸).

An obvious explanation for these differences observed between data-collection temperatures is the inhibited protein dynamics imposed by the glassy matrix at CT. At this temperature, large-scale movements are halted and only limited local atom rearrangements are possible, leading to strained geometries and trapped states: in hhMb, this prevents the water molecule from leaving the coordination sphere, despite clear reduction of the haem, as shown by the absorbance spectra. Concomitantly, the haem only undergoes a limited conformational shift within the pocket, whereas at higher temperatures a more substantial doming effect can be observed. For *Ac*NIR, the tricoordinated state of T2Cu, with no coordinating ligand, is also only observable at RT, with this a dead-end state for the enzyme, being unable to accommodate binding of the chemical substrate and allow turnover. This T2Cu state has never been obtained at CTs via prolonged X-ray exposure, but is achievable at RT and is similarly obtainable following soaking of crystals with chemical reductants (Murphy *et al.*, 1997 ▸; Strange *et al.*, 1999 ▸; Halsted *et al.*, 2019 ▸). Likewise, the water-coordinated Fe^2+^ state obtained by X-ray-induced reduction at CT is not physiologically relevant like the deoxyMb (Fe^2+^) state observed at RT. This means that when specific radiation damage is used as a crystallographic tool to visualize the structural consequences as a result of a redox transition, the structure of physiologically relevant states is more likely to be obtained during an MSOX series performed at RT rather than at CT. However, it is worth noting, as with the case for the T1Cu site in *Ac*NiR, these differences may not be clearly observable by structure alone in some metal sites of metal-containing systems, and thus other on-line complementary spectroscopic techniques, if applicable, can help to unearth this information.

In this study, we have shown that the orthogonal combination of X-ray crystallography and *in crystallo* UV–Vis absorption spectroscopy has allowed us to finely resolve the electronic configuration of the haem iron, while providing a correlated, detailed description of the structural changes. Moreover, we confirm that specific radiation damage to metals can be easily visualized in MSOX studies performed at RT (Horrell *et al.*, 2018 ▸), which is not obvious for chemical groups composed of lower *Z* atoms (see, for instance, Gotthard *et al.*, 2019 ▸). This can be explained by the high sensitivity of certain metal centres to X-rays, combined with their much higher electron density. Changes in electron density at the location of these atoms clearly outrun the loss of details due to global radiation damage, but eventually Fourier difference maps also become less and less clear at the end of our MSOX series on metal-containing proteins.

## Supplementary Material

PDB reference: horse-heart myoglobin, room temperature, 32.5 kGy, 9t6v

PDB reference: 260.0 kGy, 9t6w

PDB reference: cryogenic temperature, 676.8 kGy, 9t6x

PDB reference: 14.4 kGy, 9t6y

PDB reference: nitrite reductase from *Achromobacter cycloclastes*, room temperature, 14.9 kGy, 9t6o

PDB reference: 283.1 kGy, 9t6p

PDB reference: cryogenic temperature, 33.3 kGy, 9t6q

PDB reference: 1332 kGy, 9t6u

Supplementary Tables and Figures. DOI: 10.1107/S2059798326000690/gm5121sup1.pdf

## References

[bb1] Adam, V., Carpentier, P., Violot, S., Lelimousin, M., Darnault, C., Nienhaus, G. U. & Bourgeois, D. (2009). *J. Am. Chem. Soc.* **131**, 18063–18065. 10.1021/ja907296v 19950947

[bb2] Adam, V., Royant, A., Nivière, V., Molina-Heredia, F. P. & Bourgeois, D. (2004). *Structure*, **12**, 1729–1740. 10.1016/j.str.2004.07.013 15341736

[bb3] Agirre, J., Atanasova, M., Bagdonas, H., Ballard, C. B., Baslé, A., Beilsten-Edmands, J., Borges, R. J., Brown, D. G., Burgos-Mármol, J. J., Berrisford, J. M., Bond, P. S., Caballero, I., Catapano, L., Chojnowski, G., Cook, A. G., Cowtan, K. D., Croll, T. I., Debreczeni, J. É., Devenish, N. E., Dodson, E. J., Drevon, T. R., Emsley, P., Evans, G., Evans, P. R., Fando, M., Foadi, J., Fuentes-Montero, L., Garman, E. F., Gerstel, M., Gildea, R. J., Hatti, K., Hekkelman, M. L., Heuser, P., Hoh, S. W., Hough, M. A., Jenkins, H. T., Jiménez, E., Joosten, R. P., Keegan, R. M., Keep, N., Krissinel, E. B., Kolenko, P., Kovalevskiy, O., Lamzin, V. S., Lawson, D. M., Lebedev, A. A., Leslie, A. G. W., Lohkamp, B., Long, F., Malý, M., McCoy, A. J., McNicholas, S. J., Medina, A., Millán, C., Murray, J. W., Murshudov, G. N., Nicholls, R. A., Noble, M. E. M., Oeffner, R., Pannu, N. S., Parkhurst, J. M., Pearce, N., Pereira, J., Perrakis, A., Powell, H. R., Read, R. J., Rigden, D. J., Rochira, W., Sammito, M., Sánchez Rodríguez, F., Sheldrick, G. M., Shelley, K. L., Simkovic, F., Simpkin, A. J., Skubak, P., Sobolev, E., Steiner, R. A., Stevenson, K., Tews, I., Thomas, J. M. H., Thorn, A., Valls, J. T., Uski, V., Usón, I., Vagin, A., Velankar, S., Vollmar, M., Walden, H., Waterman, D., Wilson, K. S., Winn, M. D., Winter, G., Wojdyr, M. & Yamashita, K. (2023). *Acta Cryst.* D**79**, 449–461.

[bb4] Beitlich, T., Kühnel, K., Schulze-Briese, C., Shoeman, R. L. & Schlichting, I. (2007). *J. Synchrotron Rad.* **14**, 11–23. 10.1107/S0909049506049806 17211068

[bb5] Berglund, G. I., Carlsson, G. H., Smith, A. T., Szöke, H., Henriksen, A. & Hajdu, J. (2002). *Nature*, **417**, 463–468. 10.1038/417463a 12024218

[bb6] Boulanger, M. J. & Murphy, M. E. P. (2003). *Protein Sci.* **12**, 248–256. 10.1110/ps.0224503 PMC2312428 12538888

[bb7] Burmeister, W. P. (2000). *Acta Cryst.* D**56**, 328–341. 10.1107/s0907444999016261 10713520

[bb8] Bury, C. S., Brooks-Bartlett, J. C., Walsh, S. P. & Garman, E. F. (2018). *Protein Sci.* **27**, 217–228. 10.1002/pro.3302 PMC5734275 28921782

[bb9] Caramello, N., Adam, V., Pearson, A. R. & Royant, A. (2025). *J. Appl. Cryst.* **58**, 1068–1078. 10.1107/S1600576725003541 PMC12135974 40475934

[bb10] de la Mora, E., Coquelle, N., Bury, C. S., Rosenthal, M., Holton, J. M., Carmichael, I., Garman, E. F., Burghammer, M., Colletier, J.-P. & Weik, M. (2020). *Proc. Natl Acad. Sci. USA*, **117**, 4142–4151. 10.1073/pnas.1821522117 PMC7049125 32047034

[bb11] Emsley, P., Lohkamp, B., Scott, W. G. & Cowtan, K. (2010). *Acta Cryst.* D**66**, 486–501. 10.1107/S0907444910007493 PMC2852313 20383002

[bb12] Farver, O., Eady, R. R., Sawers, G., Prudêncio, M. & Pecht, I. (2004). *FEBS Lett.* **561**, 173–176. 10.1016/S0014-5793(04)00171-1 15013772

[bb13] Fransson, T., Chatterjee, R., Fuller, F. D., Gul, S., Weninger, C., Sokaras, D., Kroll, T., Alonso-Mori, R., Bergmann, U., Kern, J., Yachandra, V. K. & Yano, J. (2018). *Biochemistry*, **57**, 4629–4637. 10.1021/acs.biochem.8b00325 PMC6081253 29906115

[bb14] Fraser, J. S., van den Bedem, H., Samelson, A. J., Lang, P. T., Holton, J. M., Echols, N. & Alber, T. (2011). *Proc. Natl Acad. Sci. USA*, **108**, 16247–16252. 10.1073/pnas.1111325108 PMC3182744 21918110

[bb15] Frauenfelder, H., Sligar, S. G. & Wolynes, P. G. (1991). *Science*, **254**, 1598–1603. 10.1126/science.1749933 1749933

[bb16] Fukuda, Y., Tse, K. M., Nakane, T., Nakatsu, T., Suzuki, M., Sugahara, M., Inoue, S., Masuda, T., Yumoto, F., Matsugaki, N., Nango, E., Tono, K., Joti, Y., Kameshima, T., Song, C., Hatsui, T., Yabashi, M., Nureki, O., Murphy, M. E. P., Inoue, T., Iwata, S. & Mizohata, E. (2016). *Proc. Natl Acad. Sci. USA*, **113**, 2928–2933. 10.1073/pnas.1517770113 PMC4801246 26929369

[bb17] Garman, E. F. & Schneider, T. R. (1997). *J. Appl. Cryst.* **30**, 211–237.

[bb18] Ghosh, S., Dey, A., Sun, Y., Scholes, C. P. & Solomon, E. I. (2009). *J. Am. Chem. Soc.* **131**, 277–288. 10.1021/ja806873e PMC2629382 19053185

[bb19] Gotthard, G., Aumonier, S., De Sanctis, D., Leonard, G., von Stetten, D. & Royant, A. (2019). *IUCrJ*, **6**, 665–680. 10.1107/S205225251900616X PMC6608634 31316810

[bb20] Gudmundsson, M., Kim, S., Wu, M., Ishida, T., Momeni, M. H., Vaaje-Kolstad, G., Lundberg, D., Royant, A., Ståhlberg, J., Eijsink, V. G. H., Beckham, G. T. & Sandgren, M. (2014). *J. Biol. Chem.* **289**, 18782–18792. 10.1074/jbc.M114.563494 PMC4081921 24828494

[bb21] Halsted, T. P., Yamashita, K., Gopalasingam, C. C., Shenoy, R. T., Hirata, K., Ago, H., Ueno, G., Blakeley, M. P., Eady, R. R., Antonyuk, S. V., Yamamoto, M. & Hasnain, S. S. (2019). *IUCrJ*, **6**, 761–772. 10.1107/S2052252519008285 PMC6608623 31316819

[bb22] Hersleth, H.-P. & Andersson, K. K. (2011). *Biochim. Biophys. Acta*, **1814**, 785–796. 10.1016/j.bbapap.2010.07.019 20691815

[bb23] Hersleth, H.-P., Hsiao, Y.-W., Ryde, U., Görbitz, C. H. & Andersson, K. K. (2008). *Chem. Biodivers.* **5**, 2067–2089. 10.1002/cbdv.200890189 18972498

[bb24] Hersleth, H.-P., Uchida, T., Røhr, Å. K., Teschner, T., Schünemann, V., Kitagawa, T., Trautwein, A. X., Görbitz, C. H. & Andersson, K. K. (2007). *J. Biol. Chem.* **282**, 23372–23386. 10.1074/jbc.M701948200 17565988

[bb25] Hope, H., Frolow, F., von Böhlen, K., Makowski, I., Kratky, C., Halfon, Y., Danz, H., Webster, P., Bartels, K. S., Wittmann, H. G. & Yonath, A. (1989). *Acta Cryst.* B**45**, 190–199. 10.1107/s0108768188013710 2619959

[bb26] Horrell, S., Antonyuk, S. V., Eady, R. R., Hasnain, S. S., Hough, M. A. & Strange, R. W. (2016). *IUCrJ*, **3**, 271–281. 10.1107/S205225251600823X PMC4937782 27437114

[bb27] Horrell, S., Kekilli, D., Sen, K., Owen, R. L., Dworkowski, F. S. N., Antonyuk, S. V., Keal, T. W., Yong, C. W., Eady, R. R., Hasnain, S. S., Strange, R. W. & Hough, M. A. (2018). *IUCrJ*, **5**, 283–292. 10.1107/S205225251800386X PMC5929374 29755744

[bb60] Hough, M. A., Conradie, J., Strange, R. W., Antonyuk, S. V., Eady, R. R., Ghosh, A. & Hasnain, S. S. (2020). *Chem. Sci.* **11**, 12485–12492. 10.1039/d0sc04797j PMC8163067 34094452

[bb28] Hough, M. A., Eady, R. R. & Hasnain, S. S. (2008). *Biochemistry*, **47**, 13547–13553. 10.1021/bi801369y 19053252

[bb29] Kataoka, K., Furusawa, H., Takagi, K., Yamaguchi, K. & Suzuki, S. (2000). *J. Biochem.* **127**, 345–350. 10.1093/oxfordjournals.jbchem.a022613 10731703

[bb30] Lamb, D. C., Ostermann, A., Prusakov, V. E. & Parak, F. G. (1998). *Eur. Biophys. J.* **27**, 113–125. 10.1007/s002490050117 10950634

[bb31] Liebschner, D., Afonine, P. V., Baker, M. L., Bunkóczi, G., Chen, V. B., Croll, T. I., Hintze, B., Hung, L.-W., Jain, S., McCoy, A. J., Moriarty, N. W., Oeffner, R. D., Poon, B. K., Prisant, M. G., Read, R. J., Richardson, J. S., Richardson, D. C., Sammito, M. D., Sobolev, O. V., Stockwell, D. H., Terwilliger, T. C., Urzhumtsev, A. G., Videau, L. L., Williams, C. J. & Adams, P. D. (2019). *Acta Cryst.* D**75**, 861–877. 10.1107/S2059798319011471 PMC6778852 31588918

[bb32] Makita, H., Simon, P. S., Kern, J., Yano, J. & Yachandra, V. K. (2023). *Curr. Opin. Struct. Biol.* **80**, 102604. 10.1016/j.sbi.2023.102604 PMC10793627 37148654

[bb33] McCarthy, A. A., Basu, S., Bernaudat, F., Blakeley, M. P., Bowler, M. W., Carpentier, P., Effantin, G., Engilberge, S., Flot, D., Gabel, F., Gajdos, L., Kamps, J. J. A. G., Kandiah, E., Linares, R., Martel, A., Melnikov, I., Mossou, E., Mueller-Dieckmann, C., Nanao, M., Nurizzo, D., Pernot, P., Popov, A., Royant, A., de Sanctis, D., Schoehn, G., Talon, R., Tully, M. D. & Soler-Lopez, M. (2025). *J. Synchrotron Rad.* **32**, 577–594. 10.1107/S1600577525002012 PMC12067332 40226912

[bb34] McGeehan, J., Ravelli, R. B. G., Murray, J. W., Owen, R. L., Cipriani, F., McSweeney, S., Weik, M. & Garman, E. F. (2009). *J. Synchrotron Rad.* **16**, 163–172. 10.1107/S0909049509001629 PMC2651762 19240328

[bb35] Murphy, M. E. P., Turley, S. & Adman, E. T. (1997). *J. Biol. Chem.* **272**, 28455–28460. 10.1074/jbc.272.45.28455 9353305

[bb36] Nave, C. & Garman, E. F. (2005). *J. Synchrotron Rad.* **12**, 257–260. 10.1107/S0909049505007132 15840908

[bb37] Owen, R. L., Axford, D., Sherrell, D. A., Kuo, A., Ernst, O. P., Schulz, E. C., Miller, R. J. D. & Mueller-Werkmeister, H. M. (2017). *Acta Cryst.* D**73**, 373–378. 10.1107/S2059798317002996 PMC5379936 28375148

[bb38] Pfanzagl, V., Beale, J. H., Michlits, H., Schmidt, D., Gabler, T., Obinger, C., Djinović-Carugo, K. & Hofbauer, S. (2020). *J. Biol. Chem.* **295**, 13488–13501. 10.1074/jbc.RA120.014087 PMC7521648 32723869

[bb39] Pompidor, G., Dworkowski, F. S. N., Thominet, V., Schulze-Briese, C. & Fuchs, M. R. (2013). *J. Synchrotron Rad.* **20**, 765–776. 10.1107/S0909049513016063 PMC3747950 23955041

[bb40] Ravelli, R. B. & McSweeney, S. M. (2000). *Structure*, **8**, 315–328. 10.1016/s0969-2126(00)00109-x 10745008

[bb41] Rose, S. L., Antonyuk, S. V., Sasaki, D., Yamashita, K., Hirata, K., Ueno, G., Ago, H., Eady, R. R., Tosha, T., Yamamoto, M. & Hasnain, S. S. (2021). *Sci. Adv.* **7**, eabd8523. 10.1126/sciadv.abd8523 PMC7775769 33523860

[bb42] Rose, S. L., Baba, S., Okumura, H., Antonyuk, S. V., Sasaki, D., Hedison, T. M., Shanmugam, M., Heyes, D. J., Scrutton, N. S., Kumasaka, T., Tosha, T., Eady, R. R., Yamamoto, M. & Hasnain, S. S. (2022). *Proc. Natl Acad. Sci. USA*, **119**, e2205664119. 10.1073/pnas.2205664119 PMC9335323 35862453

[bb43] Rose, S. L., Ferroni, F. M., Horrell, S., Brondino, C. D., Eady, R. R., Jaho, S., Hough, M. A., Owen, R. L., Antonyuk, S. V. & Hasnain, S. S. (2024). *J. Mol. Biol.* **436**, 168706. 10.1016/j.jmb.2024.168706 39002715

[bb44] Schlichting, I., Berendzen, J., Chu, K., Stock, A. M., Maves, S. A., Benson, D. E., Sweet, R. M., Ringe, D., Petsko, G. A. & Sligar, S. G. (2000). *Science*, **287**, 1615–1622. 10.1126/science.287.5458.1615 10698731

[bb45] Sen, K., Horrell, S., Kekilli, D., Yong, C. W., Keal, T. W., Atakisi, H., Moreau, D. W., Thorne, R. E., Hough, M. A. & Strange, R. W. (2017). *IUCrJ*, **4**, 495–505. 10.1107/S2052252517007527 PMC5571812 28875036

[bb46] Strange, R. W., Murphy, L. M., Dodd, F. E., Abraham, Z. H. L., Eady, R. R., Smith, B. E. & Hasnain, S. S. (1999). *J. Mol. Biol.* **287**, 1001–1009. 10.1006/jmbi.1999.2648 10222206

[bb47] Stubbs, J., Caramello, N., Rodrigues, M. J., Engilberge, S., Mathieu, E., Rose, S. L., Evans, G., Royant, A. & Tews, I. (2026). Submitted. 10.1107/S2059798326002743 PMC13134000 42011725

[bb48] Taberman, H., Bury, C. S., van der Woerd, M. J., Snell, E. H. & Garman, E. F. (2019). *J. Synchrotron Rad.* **26**, 931–944. 10.1107/S1600577519005599 PMC6613113 31274415

[bb49] Vonrhein, C., Tickle, I. J., Flensburg, C., Keller, P., Paciorek, W., Sharff, A. & Bricogne, G. (2018). *Acta Cryst.* A**74**, a360.

[bb50] von Stetten, D., Giraud, T., Carpentier, P., Sever, F., Terrien, M., Dobias, F., Juers, D. H., Flot, D., Mueller-Dieckmann, C., Leonard, G. A., de Sanctis, D. & Royant, A. (2015). *Acta Cryst.* D**71**, 15–26. 10.1107/S139900471401517X PMC4304682 25615856

[bb51] Weik, M., Bergès, J., Raves, M. L., Gros, P., McSweeney, S., Silman, I., Sussman, J. L., Houée-Levin, C. & Ravelli, R. B. G. (2002). *J. Synchrotron Rad.* **9**, 342–346. 10.1107/s0909049502014589 12409620

[bb52] Weik, M., Ravelli, R. B. G., Kryger, G., McSweeney, S., Raves, M. L., Harel, M., Gros, P., Silman, I., Kroon, J. & Sussman, J. L. (2000). *Proc. Natl Acad. Sci. USA*, **97**, 623–628. 10.1073/pnas.97.2.623 PMC15380 10639129

[bb53] Yamashita, K., Wojdyr, M., Long, F., Nicholls, R. A. & Murshudov, G. N. (2023). *Acta Cryst.* D**79**, 368–373. 10.1107/S2059798323002413 PMC10167671 37158197

[bb54] Yano, J., Kern, J., Irrgang, K.-D., Latimer, M. J., Bergmann, U., Glatzel, P., Pushkar, Y., Biesiadka, J., Loll, B., Sauer, K., Messinger, J., Zouni, A. & Yachandra, V. K. (2005). *Proc. Natl Acad. Sci. USA*, **102**, 12047–12052. 10.1073/pnas.0505207102 PMC1186027 16103362

[bb55] Zárate-Romero, A., Stojanoff, V., Cohen, A. E., Hansberg, W. & Rudiño-Piñera, E. (2019). *Arch. Biochem. Biophys.* **666**, 107–115. 10.1016/j.abb.2019.03.020 30940570

