# Supplementary material for: Coupled on-line in crystallo UV–Vis absorption spectroscopy and X-ray crystallography to compare specific radiation damage in metal-containing proteins at room versus cryogenic temperature
Source: Acta Crystallogr D Struct Biol. 2026 Feb 5;82(Pt 3):187–98. doi: 10.1107/S2059798326000690 (PMC12954861; doi:10.1107/S2059798326000690)
Supplement: Supplementary file 1 [file d-82-00187-sup1.pdf]

Acta Cryst D

STRUCTURAL BIOLOGY

Volume 82 (2026)

Supporting information for article:

Coupled online *in crystallo* UV–Vis absorption spectroscopy and X-ray crystallography to compare specific radiation damage in metal-containing proteins at room versus cryogenic temperature

Nicolas Caramello, Samuel L. Rose, Eric Mathieu, Lucas Petit, Ivo Tews, Sylvain Engilberge and Antoine Royant

Table S1 Data collection and data reduction statistics – horse heart myoglobin

PDB entry	9T6y	9t6x	9t6v	9t6w
Temperature	100 K	100 K	294 K	294 K
**Data collection and reduction**				
Beamline	BM07			
wavelength (Å)	0.975			
Average dose in exposed region (kGy)	14.4	676.8	32.5	260
Resolution range (Å)	26.657 - 1.030 (1.111 - 1.030)	27.743 - 1.103 (1.122 - 1.103)	21.317 - 1.243 (1.264 - 1.243)	31.284 - 1.713 (1.743 - 1.713)
Space group	$P2_12_12_1$	$P2_12_12_1$	$P2_12_12_1$	$P2_12_12_1$
Unit cell				
a, b, c (Å)	28.471<br>35.084<br>123.11	28.484<br>35.101<br>123.16	29.106<br>35.825<br>125.32	29.187<br>35.782<br>125.11
Number of reflections				
Total	333967 (16440)	324114 (15244)	486389 (20557)	190482 (8772)
Unique	52178 (2553)	50652 (2446)	37631 (1804)	14687 (677)
Multiplicity	6.4 (6.4)	6.4 (6.2)	12.9 (11.4)	13.0 (13.0)
Completeness ellipsoidal (%)	99.8 (99.5)	99.9 (99.8)	99.3 (97.5)	99.3 (100.0)
R_{meas}	0.177 (2.643)	0.194 (3.072)	0.198 (2.26)	0.178 (2.634)
R_{pim}	0.069 (1.027)	0.076 (1.209)	0.055 (0.645)	0.049 (0.721)
$\langle I/\sigma(I) \rangle$	5.6 (0.7)	4.9 (0.6)	6.4 (1.0)	7.9 (0.9)
CC1/2	0.997 (0.379)	0.995 (0.334)	0.995 (0.632)	0.998 (0.699)

Table S2 Data collection and data reduction statistics – copper-containing nitrite reductase

**PDB entry**	**9t6q**		**9t6u**	**9t6o**		**9t6p**
Temperature	100 K	100 K	100 K	294 K	294 K	294 K
**Data collection and reduction**						
Beamline	BM07					
wavelength (Å)	0.975					
Average dose in exposed region (kGy)	33.3	666	1332	14.9	104.3	283.1
Resolution range (Å)	27.53 - 1.464 (1.489 - 1.464)	26.456 - 1.471 (1.496 - 1.471)	28.764 - 1.504 (1.53 - 1.504)	26.756 - 1.605 (1.633 - 1.605)	26.769 - 1.663 (1.692 - 1.663)	29.117 - 2.016 (2.051 - 2.016)
Space group	P$2_1$3	P$2_1$3	P$2_1$3	P$2_1$3	P$2_1$3	P$2_1$3
Unit cell						
a=b=c (Å)	95.352	95.365	95.375	96.472	96.518	96.542
Number of reflections						
Total	557048 (22408)	549547 (22059)	518155 (24839)	441991 (22202)	397656 (19764)	225028 (11597)
Unique	49834 (2426)	49167 (2376)	46178 (2339)	39308 (1939)	35448 (1747)	20090 (1013)
Multiplicity	11.2 (9.2)	11.2 (9.3)	11.2 (10.6)	11.2 (11.5)	11.2 (11.3)	11.2 (11.4)
Completeness ellipsoidal (%)	99.9 (98.1)	99.9 (98.0)	100.0 (100.0)	100.0 (100.0)	100.0 (100.0)	100.0 (100.0)
R_{meas}	0.21 (2.306)	0.2 (2.153)	0.206 (2.032)	0.248 (3.896)	0.254 (4.228)	0.417 (3.7)
R_{pim}	0.062 (0.729)	0.059 (0.679)	0.061 (0.617)	0.074 (1.148)	0.075 (1.251)	0.124 (1.089)
$<I/\sigma(I)>$	9.1 (1.0)	9.7 (1.0)	9.4 (1.2)	7.0 (0.6)	6.9 (0.6)	5.8 (0.8)
CC1/2	0.997 (0.354)	0.998 (0.4)	0.997 (0.506)	0.995 (0.333)	0.995 (0.362)	0.991 (0.35)

Table S3 Refinement statistics – horse heart myoglobin

PDB entry	9t6y	9t6x	9t6v	9t6w
Temperature	100 K	100 K	294 K	294 K
**Structure refinement**				
Resolution range (Å)	27.74 - 1.03 (1.11 - 1.03)	30.49 - 1.1 (1.18 - 1.1)	21.32 - 1.243 (1.28 - 1.24)	34.4 - 1.71 (1.84 - 1.71)
Reflections used in refinement	61719 (12090)	51035 (10021)	37434 (2676)	14708 (2858)
Reflections used for R-free	1109 (216)	998 (196)	1830 (99)	691 (126)
Solvent content (%)	32.55	32.65	37.01	37.00
Number of protein molecules in the a.u.	1	1	1	1
Number of non-hydrogen atoms				
Total	1528	1526	1428	1306
Protein	1282	1340	1261	1232
Solvent	101	58	86	43
Ligands	145	128	81	31
Average B-factor ($Å^2$)				
all atoms	13.40	13.76	17.29	29.96
Protein	12.08	12.54	16.60	29.94
Solvent	12.95	16.80	12.46	26.66
Ligands	25.45	25.15	33.08	35.25
Rwork	0.1652 (0.3586)	0.1703 (0.3136)	0.1494 (0.3410)	0.1855 (0.3285)
Rfree	0.2012 (0.4036)	0.1895 (0.3315)	0.1675 (0.3196)	0.2068 (0.3164)
RMS deviations				
on bond lengths (Å)	0.019	0.016	0.015	0.019
on bond angles (°)	1.85	1.89	1.86	1.8
Ramachandran				
favoured (%)	98.67	98.00	99.33	98.66
allowed (%)	1.33	2.00	0.67	1.34
outliers (%)	0.00	0.00	0.00	0.00
Rotamer outliers (%)	0.00	0.72	1.54	2.44
Clash score	4.04	6.39	1.48	1.97

Table S4 Refinement statistics – copper-containing nitrite reductase

PDB entry	9t6q		9t6u	9t6o		9t6p
Temperature	100 K	100 K	100 K	294 K	294 K	294 K
**Structure refinement**						
Resolution range (Å)	28.75 - 1.465 (1.49 - 1.46)	28.75 - 1.472 (1.5 - 1.47)	28.76 - 1.5 (1.53 - 1.5)	27.85 - 1.6 (1.72 - 1.6)	27.86 - 1.658 (1.7 - 1.66)	29.11 - 2.008 (2.11 - 2.01)
Reflections used in refinement	49737 (2713)	49024 (2695)	46355 (2702)	39612 (7816)	35673 (2665)	20278 (2824)
Reflections used for R-free	2493 (138)	2456 (134)	2320 (131)	1978 (368)	1785 (125)	1004 (148)
Solvent content (%)	38.40	38.26	38.45	40.52	40.79	40.65
Number of protein molecules in the a.u.	1	1	1	1	1	1
Number of non-hydrogen atoms						
total	3076	3070	3032	2851	2831	2720
protein	2621	2616	2605	2678	2674	2608
solvent	60	60	37	7	7	7
Ligands	395	394	390	166	150	105
Average B-factor (Å^2)						
all atoms	16.85	17.31	17.58	22.85	24.61	32.20
protein	14.93	15.38	15.84	22.27	24.16	32.03
Ligands	34.08	34.04	29.76	36.64	40.40	52.17
solvent	27.00	27.63	28.08	31.61	31.79	35.20
Rwork	0.1654 (0.3139)	0.1642 (0.3144)	0.1649 (0.3097)	0.1570 (0.2990)	0.1608 (0.3202)	0.1664 (0.2962)
Rfree	0.1949 (0.3090)	0.1915 (0.2876)	0.1937 (0.3602)	0.1814 (0.2930)	0.1873 (0.3060)	0.2039 (0.3196)
RMS deviations						

on bond lengths (Å)	0.012	0.012	0.012	0.01	0.01	0.01
on bond angles (°)	1.61	1.59	1.6	1.56	1.57	1.76
			Ramachandran			
favoured (%)	97.87	97.88	98.48	98.48	99.09	98.18
allowed (%)	1.82	1.82	1.52	1.52	0.91	1.82
outliers (%)	0.30	0.30	0.00	0.00	0.00	0.00
Rotamer outliers (%)	0.36	0.36	0.72	1.78	2.50	3.32
Clash score	3.41	4.36	3.84	3.39	3.96	3.32

Table S5 Coordination distances in T1Cu for copper-containing nitrite reductase

Cryogenic temperature dose series

Dose (kGy)	33.3	66.6	99.9	133.2	166.5	333	666	1332
**Coordination distance (Å)**								
T1Cu-Met150 S^{σ}	2.45	2.44	2.45	2.46	2.45	2.45	2.48	2.43
T1Cu-Cys136 S^{γ}	2.21	2.22	2.23	2.24	2.24	2.25	2.26	2.26
T1Cu-His145 $N^{\delta1}$	1.94	1.98	1.99	1.98	2.00	2.00	2.03	2.02
T1Cu-His95 $N^{\delta1}$	2.02	2.04	2.02	2.04	2.04	2.06	2.05	2.05

Room temperature dose series

Dose (kGy)	14.9	29.8	44.7	59.6	74.5	104.3	149	283.1
**Coordination distance (Å)**								
T1Cu-Met150 S^{σ}	2.53	2.56	2.54	2.55	2.53	2.54	2.55	2.57
T1Cu-Cys136 S^{γ}	2.18	2.19	2.18	2.17	2.20	2.22	2.20	2.23
T1Cu-His145 $N^{\delta1}$	1.90	1.91	1.92	1.94	1.93	1.96	1.96	2.00
T1Cu-His95 $N^{\delta1}$	2.05	2.05	2.05	2.07	2.06	2.08	2.06	2.09

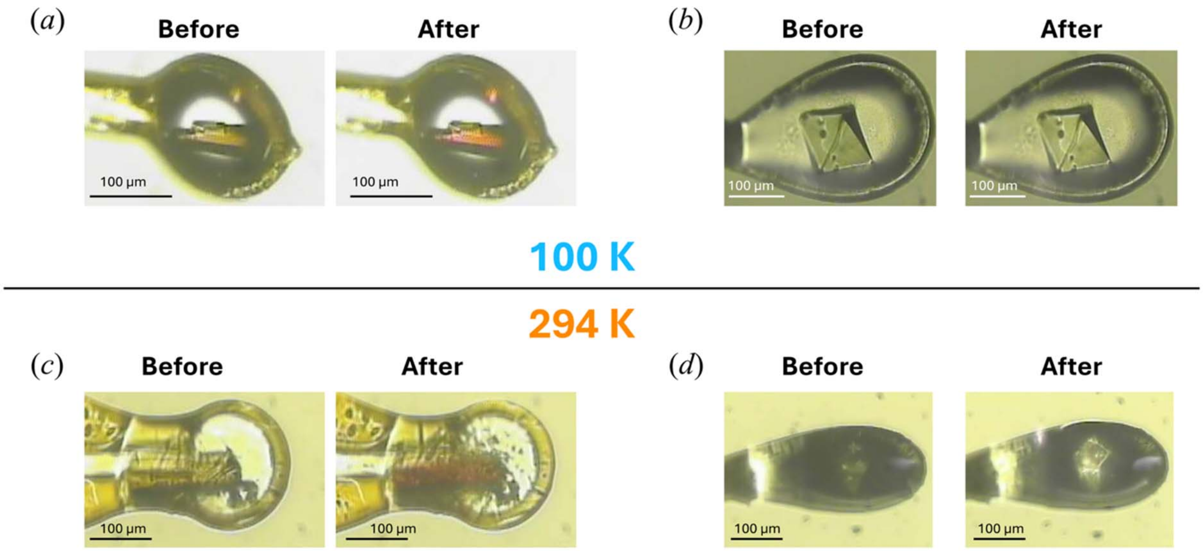

Figure S1 Snapshots of crystals before and after exposure to the X-ray beam of BM07 for (*a*) hhMb at CT (absorbed doses of 0 kGy and 676 kGy respectively), (*b*) *Ac*NIR at CT (absorbed dose of 0 kGy and1.3 MGy, respectively), (*c*) hhMb at RT (absorbed doses of 0 and 552 kGy, respectively) and (*d*) *Ac*NIR at RT (absorbed doses of 0 kGy and 1.3 MGy, respectively)

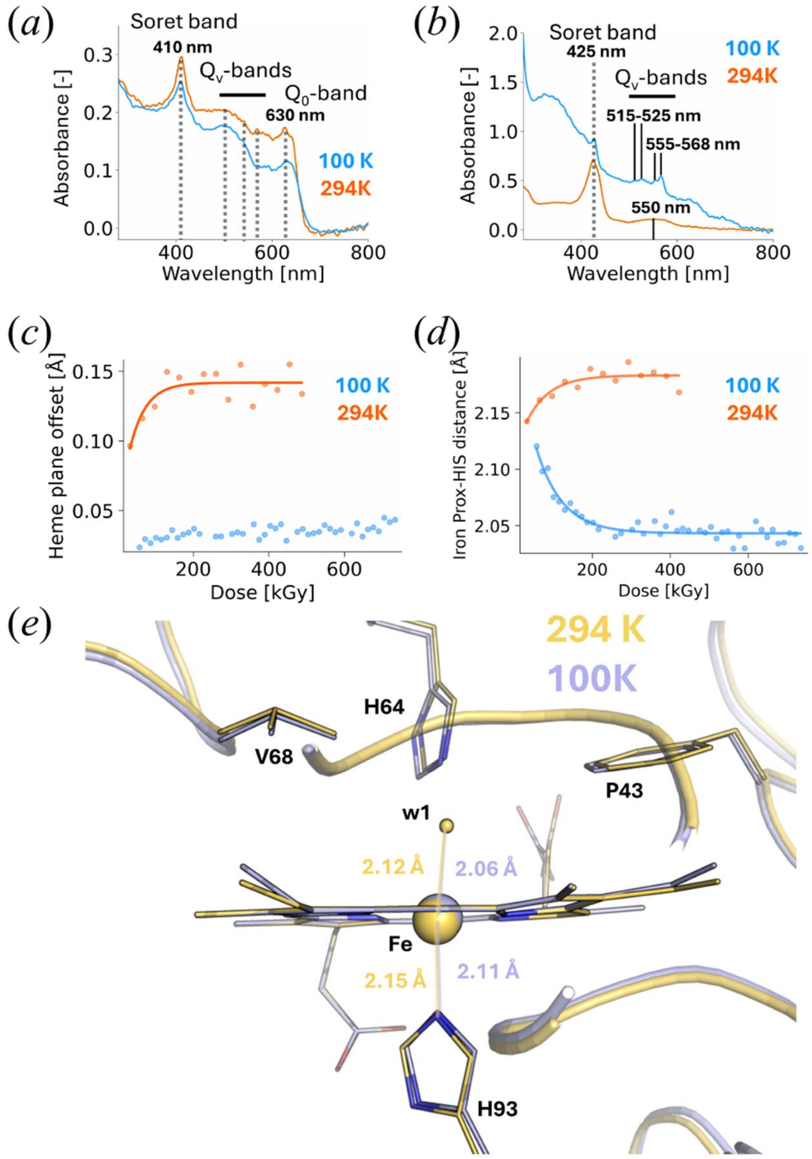

Figure S2 *In crystallo* UV-Vis absorption spectra of hhMb crystals in their (*a*) oxidised and (*b*) reduced states at CT (blue) and RT (orange). In order to make out the Soret bands in these spectra, thinner crystals were used, causing a poorer signal/noise ratio in the Q_v-band region. (*c*) Evolution of the distance between the haem iron and the plane of the haem as a function of dose at CT (blue) and at RT (orange), for which an atom displacement dose constant of 42 kGy was calculated (plain orange curve). (*d*) Evolution of the distance between the haem iron and the proximal histidine as a function of dose at CT (blue dots), for which a bond shrinkage dose constant was calculated to be 67 kGy (plain blue curve), and at RT (orange dots), for which a bond shrinkage dose constant was calculated to be 60 kGy (plain orange curve). (*e*) Refined structures of the 100 K 14.4 kGy (light blue) and 294 K 32.5 kGy (pale orange) hhMb structures (PDB entries 9t6y and 9t6v, respectively) aligned on the atoms of the haem plane only. The coordinating water molecule, haem iron and coordination distances have been coloured based on their temperature for readability.

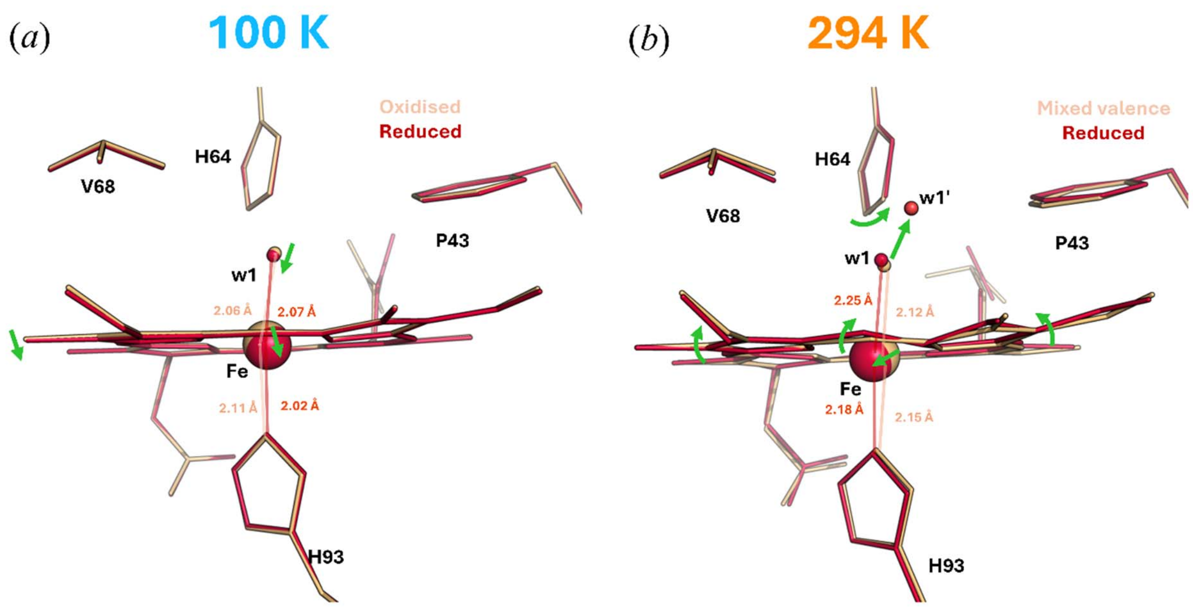

Figure S3 (*a*) Comparison of low-dose (beige) and high-dose (red) structures of hhMb at CT (PDB entries 9t6y and 9t6x, respectively), with green arrows evidencing the concerted downward movement of the haem and its coordinating water molecule at CT. (*b*) similar comparison at RT (PDB entries 9t6v and 9t6w, respectively), with green arrows evidencing the doming movement of the haem, exit of the coordinating water molecule w1 and slight rotation of His64.

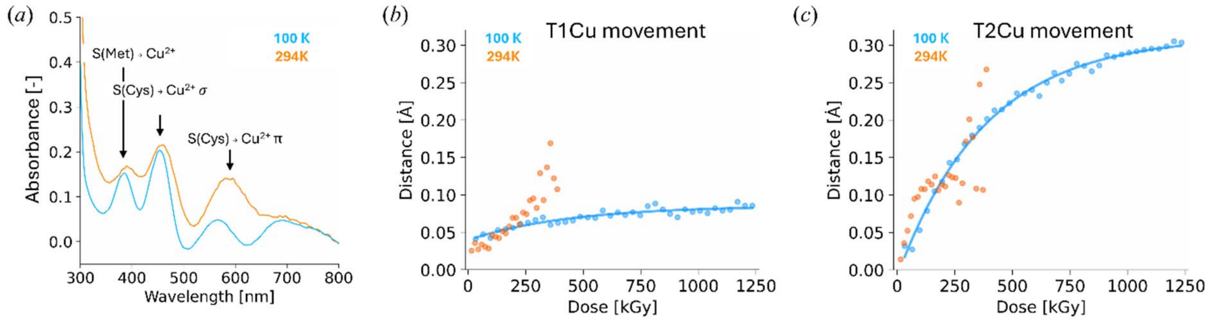

Figure S4 (*a*) *In crystallo* CT (blue) and RT (orange) UV-Vis absorption spectra of *Ac*NIR with bands assigned, showing the effect of temperature on the S(Cys)σ / S(Cys)π band ratio. (*b*) Evolution as a function of dose of the distance between the position of the Type 1 Cu compared to its position in the first dataset, increasing moderately at CT with a dose constant of 444 kGy (blue) and increasing more prominently at RT (orange, with no clear monoexponential behaviour). (*c*) Type 2 Cu dropping into the plane of His100, His135, His255, His306 at CT with a dose constant of 382 kGy (blue) and at RT (orange, with no clear monoexponential behaviour).

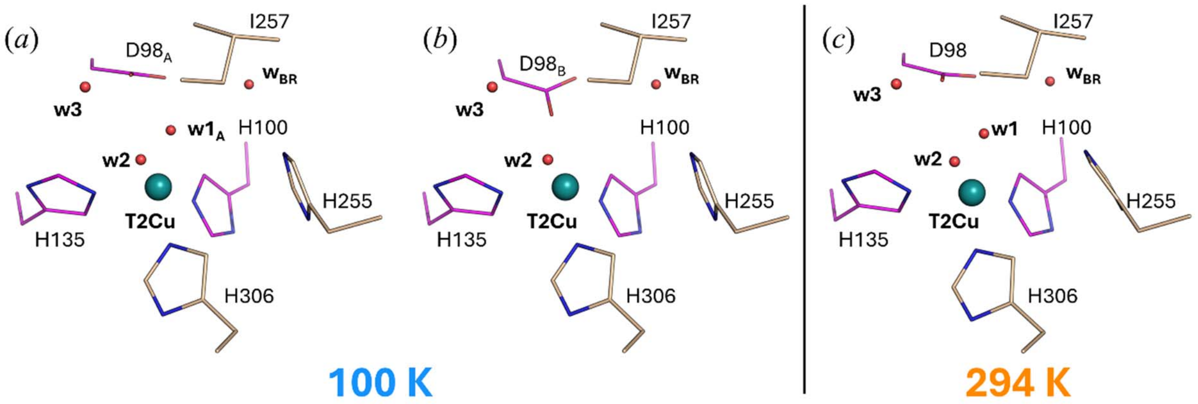

Figure S5 Active sites of *Ac*NIR represented at CT (PDB entry 9t6q), with residues of chain A represented in magenta and residues of chain B represented in beige. (*a*) First alternate conformation of the *Ac*NIR active site, with w1 populated and Asp98$_{CAT}$ out of the T2Cu coordination sphere. The bridging water molecule is marked with w$_{BR}$. (*b*) Second alternate conformation of the *Ac*NIR active site, with w1 depleted and Asp98$_{CAT}$ reaching into the T2Cu coordination sphere. The bridging water molecule is marked with w$_{BR}$. (c) Active site of *Ac*NIR represented at RT (PDB entry 9t6o), with residues of chain A represented in magenta and residues of chain B represented in beige. The active site feature only one conformation of Asp98$_{CAT}$ with w1 and w2 fully populated. The bridging water molecule is marked with w$_{BR}$.

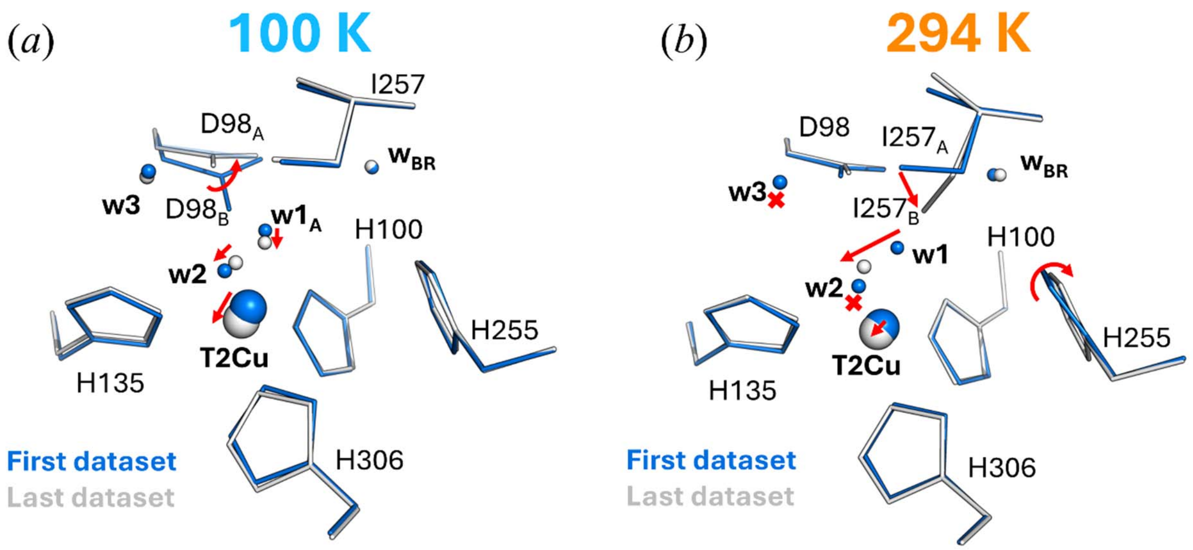

Figure S6 (*a*) Comparison of low-dose (blue coloured) and higher-dose (white coloured) *Ac*NIR active sites of *Ac*NIR at CT (refined models of PDB entries 9t6q and 9t6u, respectively), with red arrow evidencing the drop of the T2Cu into the plane of His135, His100 and His306 with concerted movements of w1 and w2, as well as the collapse of the alternate conformation of $Asp98_{CAT}$. The bridging water molecule is marked with w_{BR}. (*b*) Similar comparison of low-dose (blue coloured) and higher-dose (white coloured) *Ac*NIR active sites at RT (PDB entries 9t6o and 9t6p, respectively), with red arrows evidencing the rotation of H255 and the collapse of Ile257 into the active site, allowed by the depletion of w2 and movement of w1 (depleted w2 and w3 are marked with red crosses). The bridging water molecule is marked with w_{BR}.
